# Using Medicinal Plants in Valmalenco (Italian Alps): From Tradition to Scientific Approaches

**DOI:** 10.3390/molecules25184144

**Published:** 2020-09-10

**Authors:** Martina Bottoni, Fabrizia Milani, Lorenzo Colombo, Kevin Nallio, Paola Sira Colombo, Claudia Giuliani, Piero Bruschi, Gelsomina Fico

**Affiliations:** 1 Department of Pharmaceutical Science, University of Milan, 20133 Milan, Italy; martina.bottoni@unimi.it (M.B.); fabrizia.milani@unimi.it (F.M.); lorecolo.93@gmail.com (L.C.); kevin.nallio@studenti.unimi.it (K.N.); pasico19@virgilio.it (P.S.C.); claudia.giuliani@unimi.it (C.G.); 2 Botanical Garden G.E. Ghirardi, Department of Pharmaceutical Science, University of Milan, Toscolano Maderno, 25088 Brescia, Italy; 3 Department of Agricultural, Environmental, Food and Forestry Science and Technology, University of Florence, 50144 Florence, Italy; piero.bruschi@unifi.it

**Keywords:** Valmalenco, ethnobotany, traditional uses, medicinal plants, climate change

## Abstract

This ethnobotanical survey was carried out in Caspoggio (Valmalenco, SO, Italy) with the purpose of investigating the traditional uses of medicinal plants. Moreover, a bibliographic research meant to validate or refute the uses, focusing on the potentially responsible compounds, was performed. Fifty-nine species, attributable to 30 families (Asteraceae, Pinaceae, Malvaceae, and Lamiaceae the most cited), were mentioned. *Arnica montana*, anti-inflammatory for traumas and musculoskeletal pains; *Pinus mugo*, expectorant; *Malva sylvestris*, anti-inflammatory and soothing; *Achillea moschata*, digestive. The compounds, responsible for the therapeutic activities, are often polyphenols and terpenoids: helenanin in *A. montana*, α-pinene, δ-3-carene, and limonene in *P. mugo*, gossypin and malvin in *M. sylvestris*, luteolin and apigenin in *A. moschata*. Scientific evidence for at least one of the traditional activities described was found for 50 species but only in 26 out of 196 works consulted, it is possible to make a comparison between investigated extracts and traditional preparations. This study is thus a stimulus to new phytochemical investigations, mimicking as much as possible the traditional preparations. This work is part of the European Interreg Italy-Switzerland B-ICE project, aimed at creating a management model for the ongoing climate change and searching for new sources of territory valorization as attractions for tourists.

## 1. Introduction

Ethnobotany of mountain regions is receiving increased interest in recent years [1] due to the growing awareness about the impact of global warming on mountain biodiversity and the issues of conservation and sustainable management of the landscape. Mountain areas are recognized to be an important reservoir of ethnobotanical knowledge [2,3]. The high diversity of habitats and the historical isolation of the local communities have led to a strong diversification of the local cultural heritage. However, ethnobotanical knowledge is disappearing in mountain areas of many parts of the world, particularly in developed countries, due to the ongoing socio-economic changes and the loss of ethnic cultures. This process has been widely investigated, since loss of traditional knowledge could result in declining capacities of local communities to manage and conserve their ecosystems [4] and, therefore, has direct consequences on the use of biological resources by the next generations [5,6]. According to Dutfield [7] the erosion of ethnopharmacological knowledge and the abandonment of traditional practices might also involve a loss of access to a stock of bioactive compounds potentially useful for therapeutic application and drug development. As pointed out in many papers [7,8], traditional knowledge may be an important source of information in synthetic drug development and in natural-product research. Several bioprospection studies aiming at investigating plant-based biologically active agents have been developed in the recent years; the first step, and one of the major challenges, is the selection of plants for pharmacological studies. According to an ethnodirected approach, candidate plants can be selected by using information collected in ethnobotanical or ethnopharmacological field researches and analyzed by using quantitative tools [9]. It should be noted that traditional know-how is only a part of a large reservoir of available knowledge [7], including database, books and journal papers; information reported by informants need to be verified and validated through a comparison with the available scientific literature [7,8].

**Figure 1:**
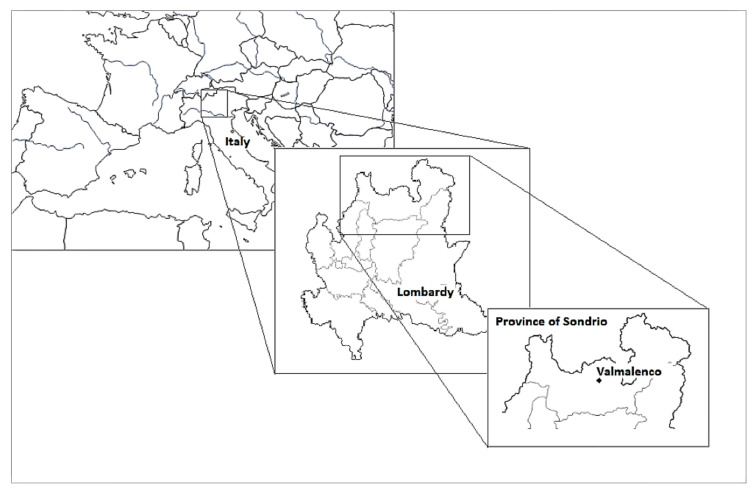
Geographical position of Valmalenco (SO, Italy).

The present study is the first ethnobotanical investigation in Valmalenco (Figure 1). It is part of the European Interreg Italy-Switzerland B-ICE project, aimed at creating a management model for the ongoing climate change and searching for new opportunities of territory valorization as attractions for tourists. Specifically, our work focused on Caspoggio, a village of about 1,400 inhabitants, located at 1098 m above sea level, enclosed by the Bernina Alps. Situated at the border with Valtellina, in the province of Sondrio (Lombardy–Italy), Valmalenco was an important link between Italy and Switzerland due to a millenary thoroughfare that used to cross the entire valley: the ‘cavallera del Muretto’ road. Between the 19th and 20th centuries, alternative passes were created and that ancient road was closed, resulting in the impoverishment of the local society, economy, and territory.

This study combines ethnobotanical field research with chemical and pharmacological information reported in books and journal articles, in order to assess the pharmaceutical value of plants traditionally used by people living in Valmalenco. Furthermore, through the rediscovering and valorization of this traditional knowledge, the present study wants to provide basic information for an efficient and sustainable approach to the use of the local natural resources.

In this research we: (1) investigated the traditional uses of plant species locally used for human and animal healthcare; (2) consulted both ethnobotanical and pharmacological literature for scientific evidence meant to validate the traditional uses reported by the informants; (3) linked plants secondary metabolites classes to the reported biological activities.

## 2. Results and Discussion

### 2.1. Field Work

A total of 137 informants were interviewed in Caspoggio. All collected data is summarized in Table S1, providing for each cited species the family, the botanical name, the vernacular name, the used plant part and preparation, the field of use, the category of use, and the detailed use. The last three columns concern the bibliographic research carried out on the species and are thus organized: Bibliographic reference: Ethnobotany (similar traditional uses throughout Italy and the world); Bibliographic reference: Biological activity (in vitro, in vivo studies or clinical trials); Bibliographic reference: Active compounds (studies on correlation between some of the active compounds of the species and their potential biological activity described in Caspoggio).

The study provides information on 59 species (1659 citations) used for human and animal healthcare, belonging to 53 genera and 30 botanical families. Asteraceae is the most cited family (692 citations for 13 species, 41.7% of the total citations), including plants both well known in the studied area (e.g., *Achillea moschata* Wulfen, *Arnica montana* L., and *Matricaria chamomilla* L.) and multi-purpose plants, e.g., used in different disease categories (e.g., *Achillea millefolium* L.). From the literature, it is evident that Asteraceae is actually the most representative family in Valmalenco (with 46 species, 27.7% of the autochthonous species cited in a reference text concerning the spontaneous plants in the province of Sondrio [10]). Other families with a high number of citations include either fewer species (Pinaceae: 289 citations for four species; Malvaceae: 142 citations for two species) or many plants most of which were less frequently cited by informants (Labiatae with 83 citations for *Thymus* spp. and few citations for the others). Of the mentioned plants, 36 (61%) were herbaceous perennials, 15 (25%) woody perennials and 3 sub-shrubs (5%). Annuals, biennials, lianas, ferns and lichens accounted for the remaining 9%. 86.9% of citations concerning the use of wild species and only 6.6% of cultivated species (*Brassica oleracea* L., *Calendula officinalis* L., *Laurus nobilis* L., *Lavandula angustifolia* Miller, *Matricaria chamomilla*, *Origanum vulgare* L., *Rosmarinus officinalis* L., *Salvia officinalis* L., and *Solanum tuberosum* L.). In 6.4% of the cases, the used species can be either wild or cultivated (*Malva sylvestris* L., *Matricaria chamomilla*, *Mentha x piperita* L.). The main used parts are flowers (21 species; 642 citations) and leaves (21 species; 301 citations) followed by the epigeous part (19; 222).

The most frequently reported method of preparation is infusion (736 citations; 44% of the total citations), both for internal and external administration (oral use 71%, compress 18%, washing 5%, footbath 1.5%, other 4.5%) followed by other preparation (e.g., the plant applied raw as poultice, exudate etc.; 376 citations; 23% of the total citations), syrup (228; 14% of the total citations), and oleolite (122; 7% of the total citations). Infusion from flowers or leaves is one of the most common methods of preparation in phytomedical practice as it is very easy to prepare and allows the extraction of a high quantity of bioactive metabolites. During our fieldwork, it was possible to acquire precious information concerning other preparations of the traditional remedies, through direct observation of the informants. As way of example, we mention the expectorant ‘syrup’, made through maceration in sugar of green pinecones of *P. mugo* kept in glass jars under the sun for 60 days, or the dandelion ‘honey’, soothing in case of sore throat, obtained after boiling the inflorescences in water and lemon juice, then covering them with sugar (Table 1). The dosage of the administered drugs and the duration of the treatment are not fixed. Rarely, the informant gave us information about the potential toxicity of some of the remedies if wrongly administered (i.e., the infusion of *Achillea millefolium* for stomachache and menstrual pains, when prepared with more than three flowers or drunk for more than 3 days in a row).

Most plants (44) are used individually while a few (15) are sometimes reported to be used in mixtures (3% of the total citations). Some examples of mixtures are: an infusion of *Achillea moschata* (epigeal part) and *Matricaria chamomilla* (flowers) to improve digestion; an infusion of *Betula* spp. (leaves), *Fraxinus excelsior* (leaves), and *Urtica dioica* (leaves) as diuretic and depurative of the urinary tract; an infusion of *Juniperus communis* (fruits), *A. moschata* (epigeal part), and *Tilia* spp. (flowers) as digestive and as sedative; an unguent of *Larix decidua* (resin) and *Pinus mugo* (resin) as anti-inflammatory and healing; an infusion of *Laurus nobilis* (leaves) and *Salvia officinalis* (leaves) as carminative; an infusion of *Pinus mugo* (green cones) and *Achillea moschata* (epigeal part) as digestive; an alcoholic macerate of *Taraxacum* spp. (flowers) and *Gentiana lutea* (roots) as digestive or a syrup of these plants against the flu; an infusion of *Thymus* spp. (epigeal part) and *Sambucus nigra* (flowers) against cough.

The traditional use of the reported plants is considered ‘still relevant’ in 96.6% of citations, ‘uncertain’ in 2.9%, and ‘past’ in 0.2%. Though in most cases these remedies are persistent nowadays, locals are aware of the declining number of individuals of some of the species. They report that the presence of the cited plants in the past was ‘very frequent’ in 88.7% of the cases and ‘quite frequent’ in 11.1%, while nowadays is ‘very frequent’ in 60.3% and ‘quite frequent’ in 36.0%. As way of example, the presence of *Achillea moschata*, *Arnica montana*, *Artemisia genipi*, and *Gentiana lutea* shifted from ‘very frequent’ in the past, to ‘quite frequent’ today. The last three have actually been in the list of endangered species at least since 2013, year of publication of the updated Red List of Italian Flora (Lista Rossa della Flora Italiana). Many a surveyed local people know about how harder and harder is to find *A. moschata* and *A. genipi* today due to the retreat of perennial snow and glaciers, which forces these two species to grow at higher altitudes, and to the indiscriminate collection by non-experts who, tearing their roots, jeopardize the balance of their communities.

Medicinal uses include 1613 citations (97.2% of the total citations), pertaining to 55 species, reported by 127 informants (93% of all the people interviewed). The most cited plants are *Arnica montana* (99 informants; 294 citations), *Achillea moschata* (93; 124), *Pinus mugo* (89; 256), *Malva sylvestris* (63; 108), *Thymus* spp. (52; 83), and *Matricaria chamomilla* (48; 98). All mentioned ailments are inserted in 14 categories which, after making some adjustments to that of Economic Botany Data Collection Standard (EBDCS) [11,12], fit best with the data acquired during the fieldwork (Table 2). Half of the species (28) are used in only one (16) or two (12) categories; 19 of them are used in a number of different categories ranging from three to five. The most versatile species are *Salvia officinalis*, *Pinus mugo*, and *Hypericum perforatum* (six categories); *Achillea millefolium* (7), *Achillea moschata* (8), and *Malva sylvestris* (12). As reported in Table S1 medicinal plants are mainly used to treat digestive tract disorders (28 species; 297 citations), respiratory tract infections (20; 348), general condition (20; 103), urinary tract diseases (20; 73), and musculoskeletal system disorders and traumas (16; 410). 

The Informant Consensus Factor (ICF) calculated for each disease category ranged from 0.65 to 1.00. As a rule, ICF values resulted to be high for uses reported by many informants, confirming that people agree on plants to be used in the treatment of common diseases. The highest values were recorded for early infancy ailments (ICF = 1.00), followed by musculoskeletal system diseases (0.96), respiratory tract infections (0.94), digestive tract disorders, and skin diseases and traumas (0.91); the lowest values for gynecological disorders, obstetric and puerperal problems (0.65). Some of the highest agreement levels were recorded for ailments reported as the most widespread for alpine communities ([3,13,14,15]). All these values were higher than those observed by Vitalini et al. [13] (ICF ranging from 0.60 to 0.88) and Cornara et al. [14] (0.38–0.83) but very similar to those reported by Dei Cas et al. [3] (0.80–0.92) and Vitalini et al. [15] (0.67–0.96). According to Heinrich et al. [16], informants’ consensus could be useful in selecting plants for pharmacological investigations. For this purpose, we identified the species with the highest agreement among the informants (FI) in each of the categories as a further helpful piece of information. For this analysis, we included the taxa cited at least five times for a given use. The species with the highest agreement in the musculoskeletal systems diseases were *Brassica oleracea* (FI = 100%; 19 citations), *Arnica montana* (92.9%; 92), *Hypericum perforatum* (85.7%; 24), *Achillea millefolium* (40.5; 15), and *Pinus mugo* (26.9%; 24). For respiratory tract infections, the species with the highest FI were *Crataegus monogyna* (100%; 5) and *P. mugo* (100%; 89), *Sambucus nigra* (91.7%; 11), and *Thymus* spp. (82.7%; 43). For digestive tract disorders: *Achillea moschata* (92.5%; 86), *Gentiana lutea* (89.2; 33), and *Artemisia genepi* (84.6; 11). For skin diseases and traumas: *Chelidonium majus* (100%; 9), *Calendula officinalis* (80.9%; 17), *Larix decidua* (66.7%; 10), and *Hypericum perforatum* (46.4%; 13). Based both on the FI values and the number of citations, other important uses in the local pharmacopoeia were *Matricaria chamomilla* as sedative and hypnotic (97.9%; 47), against abdominal pain (41.6%; 20) and as eyes anti-inflammatory (31.2%; 15), *Vaccinium myrtillus* to promote circulation (83.3%; 10) and to promote eyesight/eyes health (75%; 9), *Rosa canina* as laxative and to promote digestion (75%; 9) and as depurative (66.7%; 8), *Malva sylvestris* for treating stomachache and to promote digestion (52.4%; 33) as generic anti-inflammatory (46%; 29), and for oropharyngeal affections (22.2%; 14), *Plantago major* for treating skin traumas (50%; 10). 

**Figure 2:**
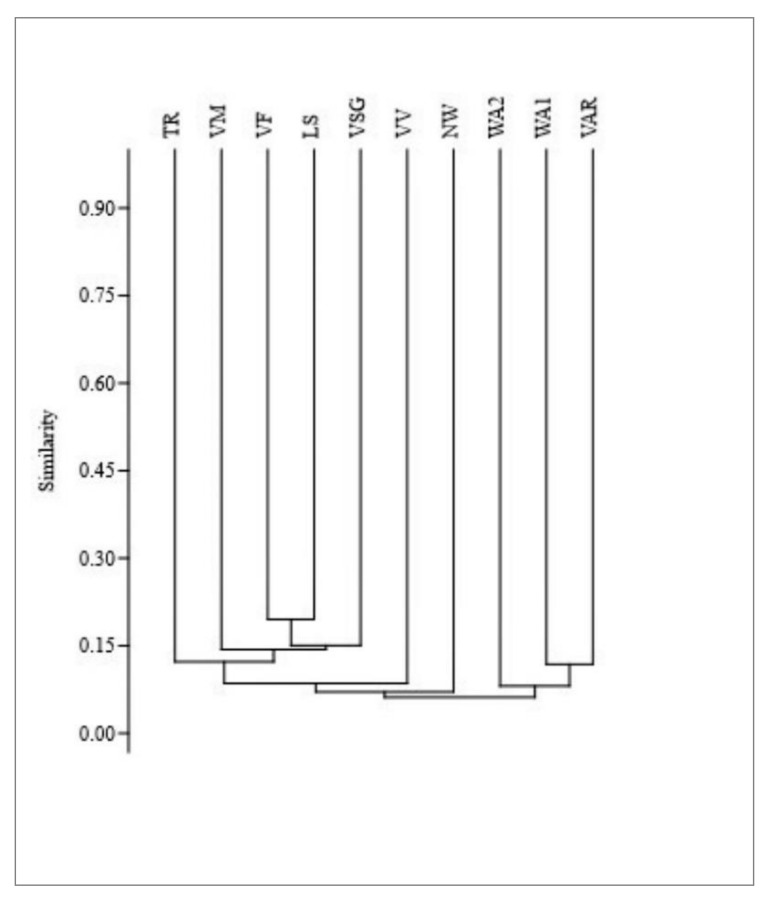
Cluster analysis of the medicinal plant use. Comparison among ethnobotanical works in Alpine and Prealpine regions. Traditional uses in Valmalenco (VM) and in neighboring areas. VV: Valvestino [19]; VSG: Val San Giacomo [13]; VF: Valfurva [3]; LS: Lombard Stelvio National Park [15]; WA1: Italian Western Alps [22]; WA2: Italian Western Alps [2]; NW: Italian North Western Alps [14]; VAR: Val Varaita [18]; TR: Tremezzina [17].

With respect to ethnobotanical studies, 11 works concerning the alpine and pre-alpine region can be found in literature [2,3,13,14,15,17,18,19,20,21,22]. Only five of them were carried out in Lombardy, in valleys surrounding Valmalenco: Valvestino (BS) [19], Val San Giacomo (SO) [13], Valfurva (SO) [3], Tremezzina (CO) [17], and in the territory of Stelvio National Park (SO) [15]. These studies point out that the most used species in folk medicine are *Achillea moschata*, *Arnica montana*, *Thymus pulegioides*, and *Artemisia genipi*. A major part of the medicinal uses (71%) recorded in these studies are specific of a certain area; 54 of the uses cited by informants in Caspoggio have not been previously reported in the consulted ethnobotanical literature of Italian Alpine areas and 53 of them are new for Italy (Table S1). These findings highlight how mountain areas are important hotspots of biocultural diversity and that different habitats as well as geographical factors and socio-cultural histories may deeply affect human relationships with the natural resources. Jaccard’s similarity index shows values ranging from 7% (Valmalenco vs. Ligurian North Western Alps) to 27% (Valmalenco vs. Valfurva) with an average value of 13% (Table 3). There is a significant negative correlation between geographic distance and the Jaccard values (Spearman: −0.876; *p* < 0.05) suggesting that villagers inhabiting areas closer to Valmalenco tend to have a similar knowledge of the use of medicinal plants. This is confirmed by a dendrogram carried out on Jaccard’s similarity index showing that ethnopharmacological data from Alpine areas of Lombardy cluster together while Piedmont and Liguria Alps group in a separated cluster (Figure 2). Valvestino (VV) seems to be an exception showing the lowest similarity value (9%) to our study area compared to the other Lombardy valleys; out of these areas, Valvestino is located at the greatest distance from Valmalenco. Based on these results it may be assumed that communities sharing ecological factors, historical backgrounds and sociocultural values have developed a similar ethnobotanical knowledge; in addition, is also possible that an exchange of ethnobotanical knowledge could have taken place, either recently or in the past, among the neighboring valleys.

Veterinary uses include 46 citations (2.8 % of the total citations), pertaining to 15 species, reported by 20 informants (1.5% of all the informants). The amount of knowledgeable informants is rather small when compared to the 127 informants citing medicinal plants in the same community but comparable to what reported in the other studies conducted in neighboring areas ([3,15]). We can hypothesize that ethno-veterinary knowledge in the study area was once higher, but due to changing socioeconomic and cultural values is currently disappearing, and only survives in the minds of a few informants. On the other hand, the breeders prefer to vaccinate and to treat their animals with synthetic products rather than rely on traditional herbal remedies. Four species (*Carlina acaulis*, *Crocus vernus, Nepeta cataria*, and *Rumex acetosa*) have only a veterinary use while the other 11 have both animal and human medicinal uses. Veterinary categories are based on the type of animals treated. The data acquired underline that eight plant species are used in cattle, four in ‘other veterinary’ (that includes alpacas, cats, and dogs), four are inserted in remedies for which the animal is not specified. Finally, one species is used in the treatment of pigs. The most cited uses are *Carduus nutans* and *Carlina acaulis* flowers administered as food for improving milk quality (eight citations, each), an infusion of *Gentiana lutea* roots to stimulate digestion in cows (five citations) and an infusion of *Matricaria chamomilla* flowers as anti-inflammatory for the eyes (five citations). It is important to point out that 13 of the cited veterinary uses have not been previously reported for Italy (Supplementary Materials Table S1).

### 2.2. Bibliographic Research

A bibliographic research was performed to find a possible match between the traditional uses and the information reported in the scientific literature about the chemical composition and the pharmaceutical activity of the cited species. A total of 196 scientific papers were identified. For 84.7% of the reported species, we found scientific studies that could validate their medicinal use. However, for some species (i.e., *Vaccinium myrtillus* and *Valeriana officinalis*) we found conflicting evidence. Species with a higher number of citations, number of informants, and number of use categories in this research have more often been subjected to scientific studies (Spearman’s Test: 0.456, *p* < 0.05; 0.443, *p* < 0.005; 0.481, *p* < 0.05). However, most of the medicinal species cited in Caspoggio have seldom been studied.

We present the comparison with scientific literature (for the complete data, please see Table S1): *Achillea millefolium* digestive, anti-inflammatory for several problems (stomachache, muscular pain, rash) and disinfectant [19,23,24,25]; *Achillea moschata* antioxidant and antibacterial [26]; *Alchemilla vulgaris* anti-inflammatory [27,28]; *Arnica montana* anti-inflammatory for wounds, cutaneous traumas, muscular, and articular pain [29,30,31,32,33,34,35,36]; *Artemisia absinthium* digestive [31,37,38,39] (however, in a review from 2016 the potential antiulcer effect, with the decrease in the production of gastric juice, is reported [40]); *Artemisia genipi* digestive and colds [39,41]; *Betula pendula* diuretic [42]; *Calendula officinalis* anti-inflammatory and disinfectant for wounds, rashes, and burns, improves circulation [31,43,44,45,46,47,48]; *Carduus nutans* antimicrobial and anti-inflammatory [49]; *Cetraria islandica* expectorant [50,51,52] and anti-inflammatory [51,53]; *Chelidonium majus* skin tags and warts [54,55]; *Cichorium intybus* depurative of the urinary tract [56,57]; *Epilobium* spp. anti-inflammatory [58,59], depurative of the urinary tract [59]; *Equisetum arvense* diuretic [60,61,62], anti-inflammatory for contusions [60,61,63,64,65,66], and analgesic [61,63,65]; *Euphrasia officinalis* eye inflammation [31,67,68]; *Foeniculum vulgare* anti-inflammatory, antispasmodic, and carminative [29,31,69,70]; *Gentiana lutea* digestive [29,31,37,71]; *Hipericum perforatum* soothing and anti-inflammatory for skin rashes and burns [23,31,72], muscular pain and contusions [72,73], and antidepressant [23,31,73,74,75,76]; *Juniperus communis* digestive [77,78,79]; *Lamium album* subsp. *album* diuretic [80,81]; *Lavandula angustifolia* anti-inflammatory and disinfectant for wounds [29,82,83,84,85], sedative [29,31,86]; *Larix decidua* anti-inflammatory and disinfectant for wounds and skin problems [31,87,88], antimicrobial [88]; *Laurus nobilis* gastroprotective [89,90,91] and menstrual pains [92,93]; *Linum usitatissimum* improves intestinal motility [94,95,96], expectorant, and decongestant [97]; *Malva sylvestris* gingivitis and inflammations of the oral cavity [98,99,100,101], demulcent, skin anti-inflammatory and regenerative [100,101,102,103,104,105], depurative of the urinary tract [106,107], and vaginal inflammation [101]; *Matricaria chamomilla* soothing and ocular anti-inflammatory [31,67], antispasmodic for the gastrointestinal tract [31,43,108,109,110], and sedative [31,43,108,109,111,112]; *Melissa officinalis* menstrual pains [113,114]; *Mentha x piperita* anti-inflammatory [115,116,117]; *Origanum vulgare* anti-inflammatory [118,119,120,121]; *Passiflora* spp. sedative hypnotic [122,123,124,125,126]; *Plantago major* anti-inflammatory and disinfectant for skin ailments [127,128,129,130], cough [127,129,130], anti-inflammatory and infections [127,128,129]; *Picea abies* gingivitis and inflammation of the oral cavity [88,131,132] and cough [31,88]; *Pinus cembra* cough and sore throat (antimicrobial) [133]; *Pinus mugo* anti-inflammatory [134], disinfectant [134], decongestant and expectorant [135,136]; *Polypodium vulgare* laxative [137]; *Prunus avium* diuretic [138]; *Rosa canina* diuretic and depurative of the urinary tract [139], decongestant and anti-inflammatory for sore throat [139,140]; *Rosmarinus officinalis* liver depurative [141,142,143,144,145,146]; *Rumex alpinus* disinfectant for wounds [147,148]; *Salvia officinalis* gingivitis [149,150], gastrointestinal bloating [150], and anti-inflammatory [150,151,152]; *Sambucus nigra* laxative [153], bronchitis, and other ailments of the upper airways [154,155,156]; *Silybum marianum* hypolipidaemic, anti-atherosclerosis [157] and hepatoprotective [23,31,157,158]; *Solanum tuberosum* anti-inflammatory [159,160,161]; *Taraxacum* spp. sore throat [162,163], skin inflammation [163], digestive and prokinetic [163,164,165], kidney problems [163,165,166,167], and anti-inflammatory [163,165,168]; *Thymus* spp. hypolipidaemic [144], balsamic and expectorant [169,170,171,172], and anti-inflammatory [170,171]; *Urtica dioica* hypolipidaemic, anti-atherosclerosis, hepatoprotective, and diuretic [173,174], anti-inflammatory, rheumatic pains, and contusions [65,173,174,175,176]; *Vaccinium myrtillus* maintenance of ocular health and function [146,177,178,179,180,181,182,183,184] (this last activity is refuted by a systematic review from 2004 [185]), improving of blood circulation [146,178,183,186], and maintaining of urinary tract health [187]; *Vaccinium vitis-idaea* maintaining of ocular health and function [180,181] and maintaining of urinary tract health [183,187,188]; *Valeriana officinalis* sedative hypnotic [189,190,191,192,193,194,195], though a systematic review from 2015 underlined no significant variation between the valerian extract and the placebo [196].

No scientific evidence was found in literature concerning the activities emerged during the survey and the following species: *Brassica oleracea* (articular pain and inflammation, decongestant for upper airways), *Carlina acaulis* subsp. *acaulis* (galactagogue and throat depurative for cattles), *Crocus vernus* (simil intoxicated state in cats after ingestion), *Fraxinus excelsior* (diuretic leaves infusion); *Mentha longifolia* (tonic and corroborant), *Oxalis acetosella* (digestive); *Nepeta cataria* (given purposely by the mother cat to her kittens); *Rumex acetosa* (improves milk and its taste if given to cows); *Tilia* spp. (digestive, antitussive).

In accordance with the evidence, potential correlations among ethnobotanical uses of a representative species, its phytochemistry, and its pharmacological properties are discussed hereafter, with the purpose to carry out a preliminary assessment on the validation of traditional uses. The case of *Pinus mugo* Turra was selected based not only on its importance in Valmalenco for frequency of citations and peculiarity of its preparations, but also on the extent and relevance of information found in scientific literature. 

### 2.3. Pinus mugo *Turra*

Traditional uses in Valmalenco attribute to *Pinus mugo* activities on integumentary, digestive, musculoskeletal, and respiratory systems, as well as veterinary purposes. Among the most cited properties, we found anti-inflammatory and disinfectant, especially for skin traumas or the treatment of cattle paws and hooves. Burns, frostbites, rushes, skin eruptions, and sprains are considered pathological conditions treatable with this species, in addition to activities such as digestive, expectorant, and decongestant, useful in the treatment of sore throat, cough, and colds. Different parts of the plant, as well as different ways of administration are used with the purpose of reaching the desired effects. Among the former are resin, sprouts, and cones. Among the latter, infusions, ointments, syrups, or the direct application, especially for the resin. In this regard, a peculiar use is referred in case of bones fractures: the fresh resin is melted, filtered, and applied directly on the fractured limb. Once hardened, it makes a natural plaster cast, functional if kept for 30–40 days. The inhabitants of the valley believe that the idle muscles are maintained strong and tonic by the resin, while a common plaster cast would cause their weakening.

Other ethnobotanical surveys in neighboring valleys refer to the use of the infusion of *P. mugo* as digestive, and of its syrup as expectorant [3,13,15,19]. From a pharmacological and phytochemical point of view, there are few studies in literature that can support this use and they all are based on characterization and evaluation of biological activity of the essential oil, often obtained from different plant matrices and from different geographic origins [135,197,198].

In particular, one of the studies discusses three EO profiles, respectively obtained from needles, twigs, and cones of *P. mugo*, as well as their anti-inflammatory and cytotoxic activities [134]. 

The anti-inflammatory activity was evaluated through the quantification of IL-6 cytokine secretion by LPS-stimulated murine macrophages, while the cytotoxic effect was tested through a viability assay of three different cancer cell lines after adding the EO.

In this study, the EOs from twigs and cones of *P. mugo* showed a significant anti-inflammatory activity compared to the other two species of *Pinus* investigated, while the EOs from needles and twigs asserted a strong cytotoxic activity on cancer cells. The former activity could be attributed to the high concentration of α-pinene in the EOs. This molecule is actually considered responsible for the inhibition of the secretion of several pro-inflammatory cytokines. Moreover, other major compounds, such as limonene and δ-3-carene, could contribute to the down-regulation of neutrophil, resulting in a reduction of the inflammatory response.

Furthermore, hydrogen peroxide, found in the EO of adult individuals of *P. mugo*, could help prolong the holding time of antimicrobial agents on the application site.

On the other hand, cytotoxic activity could be attribute to α-pinene, β-pinene, germacrene D, and α-terpinol. Among these, α-pinene seems to be responsible for the antiproliferative activity on human breast cancer cell line MCF-7 [134].

In light of the above, it is thus possible to justify the beneficial effects of *P. mugo* in several ailments described in Valmalenco. Therefore, inflammatory conditions can be attributed to tissue and vascular reactions caused by skin traumas, burns, rushes, skin eruptions, and sprains that can plausibly lead to the production of inflammatory exudates, as well as heat, redness, and pain at an epithelium level.

The anti-inflammatory effect can be considered in case of skin wounds, applicable also in the veterinary fields, where a purulent exudate can lead to a responsible infective agent.

The expectorant and decongestant properties, useful against sore throat, cough, and colds, can be as well explained through the anti-inflammatory activity explicated on the upper airways mucosal membranes. Some of the studies acknowledge to the EO of *P. mugo* antimicrobial action against fungi, yeasts, gram-positive, and gram-negative bacteria [197], especially respiratory bacteria such as *Klebsiella*, *Morganella*, *Staphylococcus* and *Escherichia* [135], as well as secretolytic effects [136], which can improve the air flow through the airways.

All the EOs tested in these studies show δ-3-carene and α-pinene as major compounds to which, synergistically with minor ones, the antimicrobial activity can be supposedly ascribed.

Therefore, these effects can be involved, together with the anti-inflammatory activity, in the management of respiratory problems or skin wounds. Nevertheless, it is of utmost importance to underline that the activities described in literature are attributed to the EO obtained from the species while, in our case, the traditional preparations used are of a completely different nature. 

Works such as ours gain value and importance in these observations, but they must be given prompt response by phytochemical and pharmacological studies, with the purpose of investigating traditional preparations and extracts and their potential biological activity.

No evidence was found concerning the digestive effects, nor the beneficial activity of the resin compared to the common plaster cast.

The bibliographic research highlighted that data on the composition of secondary metabolites, potentially related to biological activities, can be detected for 45 species out of 59. For some of these, such as *Achillea moschata*, *Artemisia absinthium*, *Linus usitatissimum*, and *Pinus mugo*, there is extensive data in respect of the different extractive methods and separation techniques, while for other species the information is lacking.

It is necessary to underline that very few extracts studied in literature can be compared to the traditional preparations cited in Caspoggio (only 26 out of the 196 studies consulted, related to 18 species. For more information, please see Table 4). For this reason, it is extremely hard to make assumptions on the involvement of isolated compounds in the biological activity described.

Ultimately, for 14 species (*Brassica oleracea*, *Carlina acaulis* subsp. acaulis, *Cichorium intybus*, *Crataegus monogyna*, *Crocus vernus*, *Fraxinus excelsior*, *Lamium album*, *Melissa officinalis*, *Mentha longifolia*, *Nepeta cataria*, *Oxalis acetosella*, *Prunus avium*, *Rumex acetosa* and *Tilia* spp.) no information was found about the correlation between their chemical characterization and the biological activity to which the traditional uses can be ascribed. Some of these, such as *B. oleracea* (cataplasm for arthritis), *C. monogyna* (upper airways infections), or *M. officinalis* (externally applied against menstrual pains), are characterized by peculiar preparations and traditional uses that require more consideration. 

## 3. Materials and Methods 

The research required the following steps:

- Preliminary Investigation on the Territory and the Local Flora

The present survey was carried out in the village of Caspoggio, in the territory of Valmalenco, a tributary valley of the Valtellina, in the province of Sondrio (Lombardy, Italy). An early bibliographic research concerning the typical local species was performed through the consultation of botany textbooks focusing on the flora of the valleys of Sondrio [10]. Due to this preliminary investigation, a table containing information on the autochthonous species (family, Latin name, common name, and habitat) was produced [200]. We supported this list with a collection of images, which could be consulted by the informants, with the purpose of helping species identification.

- Interview Management and Supporting Material

Opened and semi structured interviews were conducted through a questionnaire, consisting of an “Informant Sheet” and a “Species Sheet”. In particular, the “Informant Sheet” was split into two sections: the first one dedicated to the informant (personal data, location of the interview, education, and job), the second one focused on the interview (duration, number of people present, level of empathy, and any other useful notes).

The “Species Sheet” consisted of a 7-column table arranged as follows: Species (common and vernacular name), Field of use, Detailed use, Preparation form, Administration form, Part of the plant, Other information.

Each “Informant Sheet” was matched to a corresponding “Species Sheet”. Both were given the same one-to-one alphanumeric identification code. The interviews were thus successively filed in chronological order. 

For each species, information was organized according to the Field of use (medicinal, veterinary, etc.) and, eventually, in more detailed categories. For example, the medicinal field was divided into several categories, one for every anatomical apparatus (Digestive tract disorders, Musculoskeletal system disorders and traumas, etc.).

- Collection of Plant Material, Production of Herbarium Samples, and Photographic Archive

During the interviews and the excursions, plant material samples and traditional preparations (liquors, syrups etc.) were collected and catalogued. Plant material was dried and used for the production of herbarium samples that were archived as further support material for species identification. With the same purpose, every step of the work was documented through photographs, conveniently catalogued.

- Data Archiving and Analysis

All data obtained from the interviews were filed in a database, an Excel spreadsheet (Microsoft, Redmond, WA, USA) where each row represents a citation, defined as a single use reported for a single species by a single informant [201]. Each informant was archived with the one-to-one identification code cited above. 

Citations were considered as “distinct”, if differing from one another in at least one of the following properties: species, informant, category of use, part of the plant, preparation, and administration form. The standardization in the compilation of the database simplified both data browsing and statistical elaboration.

Data tables were derived from this worksheet using the program “EBTools”, a collection of Visual Basic for Applications (VBA) scripts executed within the Microsoft Excel framework. The scripts are designed to perform advanced data sorting, filtering, and counting operations according to specific user requirements.

The Informant Consensus Factor (ICF) [202] indicates the agreement degree among the informants (the closer the index is to 1, the higher is the agreement degree) concerning the use of species for the treatment of ailments of different organ systems. 

It was calculated as follows:

ICF = (nur − nt)/(nur − 1)(1)

where nur is the number of citations in each category; nt is the number of species used.

Informants’ consensus on medicinal uses was calculated for each species with the Fidelity Level index (FL) reported by Friedman in 1986 [203]. It was calculated as follows:

Fl = Np/N × 100(2)

where Np is the number of informants who reported the use of a species to treat a particular ailment, and N is the total number of informants who mentioned the plant for any other disease.

We calculated Jaccard’s similarity index (JI) to compare data reported in our study with previously published data collected from neighboring areas based on presence/absence of the reported uses. We focused the analysis only on uses for human health because veterinary uses were not reported in all the other considered studies. The following formula was applied:

JI = c/(a + b − c) × 100(3)

where c is the number of species common to the two sites, a is the number of species used only in the site A, and b is the number of species used only in the site B. 

A cluster analysis was finally conducted on these data by using the PAST 3.32 software package for Microsoft Windows. 

### Scientific Confirmation

Finally, an ethnobotanical, pharmacological, and phytochemical bibliographic research was carried out about species used for human and animal healthcare, through search engines and online database such as PubMed, MedLine, Google Scholar, and JANE.

Concerning the ethnobotanical research, the strategy was to combine the scientific or English common name of the species and the keywords ‘ethnobotany’, ‘ethnopharmacology’, ‘traditional medicine’, or ‘folk medicine’.

Regarding the pharmacology and phytochemistry, we paired the plant name with specific keywords concerning the category of use cited in Valmalenco (i.e., *Achillea moschata* digestive system, *Pinus mugo* respiratory system, *Arnica montana* musculoskeletal system) and then the specific pathology or activity (i.e., *Achillea moschata* digestive, *Pinus mugo* cough, *Arnica montana* anti-inflammatory, *Arnica montana* inflammation etc.).

We focused our attention particularly on systematic reviews and meta-analysis, when possible, or on single in vitro, in vivo, and clinical trials studies, applying no time filters. Based on this data, specific and detailed tables were produced (Table 4 and Supplementary Materials Table S1 [2,3,13,14,15,16,17,18,19,20,21,22,23,24,25,26,27,28,29,30,31,32,33,34,35,36,37,38,39,40,41,42,43,44,45,46,47,48,49,50,51,52,53,54,55,56,57,58,59,60,61,62,63,64,65,66,67,68,69,70,71,72,73,74,75,76,77,78,79,80,81,82,83,84,85,86,87,88,89,90,91,92,93,94,95,96,97,98,99,100,101,102,103,104,105,106,107,108,109,110,111,112,113,114,115,116,117,118,119,120,121,122,123,124,125,126,127,128,129,130,131,132,133,134,135,136,137,138,139,140,141,142,143,144,145,146,147,148,149,150,151,152,153,154,155,156,157,158,159,160,161,162,163,164,165,166,167,168,169,170,171,172,173,174,175,176,177,178,179,180,181,182,183,184,185,186,187,188,189,190,191,192,193,194,195,196,197,198,199,204,205,206,207,208,209,210,211,212,213,214,215,216,217,218,219,220,221,222,223,224,225,226,227,228,229,230,231,232,233,234,235,236,237,238,239,240,241,242,243,244,245,246,247,248,249,250,251]).

## 4. Conclusions

Documenting traditional medicinal uses can provide valuable information on locally available species which can act as potential sources of new drugs or natural products. This is particularly true in areas understudied from this perspective like the Alpine communities where our study has been carried out. Alpine regions have an environmentally complex mosaic of different habitats as well as historical and cultural peculiarities that have marked and shaped the development of a rich ethnobotanical knowledge. Through a wide survey involving 137 informants, the present investigation shows that traditional knowledge is still quite rich and alive in Valmalenco, and that medicinal plants continue to play an important role for the local communities as part of their ancient cultural heritage. The high number of traditional uses (67) not previously documented for Italian Alpine areas provide evidence of this. Furthermore, the traditional use of medicinal plants cited is considered ‘still relevant’ in 96.6% of citations, suggesting that medicinal uses are still practiced and transmitted within the community. 

Results of the pharmacological and phytochemical bibliographic research show that the scientific information for the medicinal use of the plants cited in the ethnobotanical investigation is limited. Specifically, little is found about the forms of preparation or quantities used; even when this information is available the extracts analyzed are too often very different from the typical preparations of folk medicine. Thus, it is difficult to confirm or validate traditional uses with laboratory experimentation. Moreover, some of the species reported in the present work have never been investigated from this perspective. A summary of these results is reported in Table S1 and we believe this could provide useful information on what already exists from ethnobotanical and phytochemical studies. This represents an encouragement to new investigations in this direction, meant to extend our current knowledge, with the purpose of validating ancient traditional uses.

Lastly we are convinced that a tourism valorization of the local traditional knowledge can be created and made tangible through innovative educational and leisure activities (for example, through the establishment of a botanical garden where to cultivate and to show the medicinal plants traditionally used by the local community; workshops introducing the identification of local medicinal plants or the methods of preparing creams and ointments with the herbs; treasure hunts involving children in the discovery of herbs, etc.). This approach can contribute to the safeguarding, promotion, and development of an intangible cultural heritage and at the same time represents a valuable and sustainable complement of the local touristic offering. 

## Figures and Tables

**Table 1:** Some of the traditional preparations in the village of Caspoggio.

Species	Preparation	Traditional Recipe
*Arnica montana* L. subsp. *montana* Arnica	Inflorescences exudate	Put the inflorescences in an empty glass flagon. Keep it under the summer sun. Turn the flagon upside down in order to help the exudate to leak from the inflorescences, regularly gathering the liquid.
*Calendula officinalis* L. Calendula	Macerated oil	Fill a glass jar to the brim with inflorescences. Cover the flowers with almond oil. Keep the jar under the sun for 20–30 days. Filter the macerated oil.
*Hypericum perforatum* L. Hypericum	Macerated oil	Fill a glass jar to the brim with inflorescences collected in June–July. Cover the flowers with almond, olive, or sunflower oil. Keep the jar under the summer sun. Filter the macerated oil in September.
*Larix decidua* Mill. Larch	Ointment	Mix 1 Kg of spruce and larch resin with 100g of butter, olive oil, and bee wax until it reaches a smooth texture.
*Picea abies* (L.) H.Karst Spruce		
*Pinus mugo* Turra Mountain pine	Pinecones syrup	Put green pinecones collected in June in a glass jar until they reach the brim. Cover the pinecone completely with white sugar. Keep the jar under the summer sun for 60 days, shaking it at intervals. Filter the syrup.
*Taraxacum* spp. Dandelion	Inflorescences syrup	Put 100 flower heads in a glass jar and cover them with 100 g of white sugar. Keep the jar under the summer sun for 30–40 days, until the sugar melts. Filter the syrup.

**Table 2:** Categories of use (pathologies treated in Caspoggio).

Category of Use (Pathologies)	n. Species per Category	n. Citations per Category	ICF
Digestive tract disorders	28	297	0.91
Respiratory tract infections	20	348	0.95
General condition	20	103	0.81
Urinary tract disorders	20	73	0.74
Musculoskeletal system disorders and traumas	16	410	0.96
Skin diseases and traumas	14	140	0.91
Nervous system disorders	12	97	0.89
Circulatory system disorders	12	37	0.69
Gynecological disorders, obstetric and puerperal problems	8	21	0.65
Ophthalmic ailments	7	36	0.83
Other	7	19	0.67
Oropharyngeal cavity affections	6	25	0.79
Early infancy ailments	1	6	1.00
Afflictions of the ear	1	1	

**Table 3:** Comparison among ethnobotanical works in Alpine and Prealpine regions. Jaccard Similarity Index of medicinal plant uses (veterinary excluded) in Valmalenco (VM) and in neighboring areas. VV: Valvestino [19]; VSG: Val San Giacomo [13]; VF: Valfurva [3]; LS: Lombard Stelvio National Park [15]; WA1: Italian Western Alps [22]; WA2: Italian Western Alps [2]; NW: Italian North Western Alps [14]; VAR: Val Varaita [18]; TR: Tremezzina [17].

Comparisons		Uses Reported in Both Groups	Uses Reported in One Group Only (Group 1/Group 2)	Jaccard Index	Reference	Specific Uses in Valmalenco
VM	VV	19	162/66	0.09	[19]	162
VM	VSG	41	140/133	0.18	[13]	140
VM	VF	69	112/209	0.27	[3]	112
VM	LS	89	92/539	0.16	[15]	92
VM	WA1	17	164/53	0.08	[22]	164
VM	WA2	22	159/130	0.08	[2]	159
VM	NW	24	157/198	0.07	[14]	157
VM	VAR	15	166/38	0.08	[18]	166
VM	TR	48	133/181	0.18	[17]	133
			Average	0.13	Average	142.78
					Standard deviation	26.11

**Table 4:** Comparable extracts mentioned in literature with the traditional preparations.

Latin Name	Traditional Uses in Valmalenco				Type of Preparation in Literature	Reference
	Part of the Plant and Preparation	Field of Use	Category of Use	Detailed Use		
*Achillea millefolium* L.	Epigeal part (Whole) (compresses with infusion)	Med	Skin diseases and traumas	Anti-inflammatory, disinfectant and wound healing, Emollient, soothing	Hot water extract with hemostyptic activity in vitro	[25]
					Aqueous extract with anti-inflammatory activity in vitro	[24]
*Matricaria chamomilla* L.	Flowers/inflorescences/flowering tops (infusion)	Med	-	Anti-oxidant	Aqueous extract with anti-oxidant activity in vitro	[108]
	Flowers/inflorescences/flowering tops (infusion)	Med	Nervous system disorders	Sedative hypnotic, promotes sleep	Lyophilized aqueous extract with activity on CNS	
*Taraxacum* spp.	Flowers/inflorescences/flowering tops (infusion)	Med	Urinary tract disorders	Kidney stones Cystitis and other inflammation of the urinary tract Urinary tract depurative Diuretic	Aqueous roots extract with diuretic activity in rats	[167]
	Leaves (infusion or decoction)					
	Epigeal part (Whole) (infusion)					
	Underground organs (roots/bulbs/tubers/rhizomes) (infusion or decoction)					
	Leaves (infusion or decoction)	Med	-	Anti-inflammatory	Aqueous leaves extract with anti-inflammatory activity in vivo	
*Gentiana lutea* L. subsp. *lutea*	Underground organs (roots/bulbs/tubers/rhizomes) (decoction, grappa)	Med	Digestive tract disorders	Digestive Stomach anti-inflammatory Liver anti-inflammatory Stomachache Vermifuge	EtOH roots extract with choleretic activity (not the Aqueous nor the MeOH extracts)	WHO Monograph—Radix Gentianae Luinfusione [29]
*Hypericum perforatum* L.	Flowers/inflorescences/flowering tops (macerated oil)	Med	Skin diseases and traumas	Sunburns, burns, frostbites, redness and rash Anti-inflammatory, disinfectant and wound healing Psoriasis Insect bites	Different types of macerated oils active on burns and wounds	[72]
					21 macerated oil samples of H. (homemade or commercial) analyzed. Pseudohypericin and hypericin in all samples. Hyperforin in 4 samples.	
	Flowers/inflorescences/flowering tops (macerated oil)	Med	Musculoskeletal system disorders and traumas	Contusions Sprains and dislocations Articular pain and inflammations Muscle inflammations and pain		
*Origanum vulgare* L.	Epigeal part (Whole) (infusion/compresses with infusion)	Med	-	Antioxidant activity	Antioxidant activity of hot and cold water extracts	[121]
*Rosmarinus officinalis* L.	Epigeal part (Whole) (infusion)	Med	Digestive tract disorders	Liver depurative	Choleretic and Hepatoprotective activity of aqueous extracts in rats	[141]
					Hepatoprotective and antioxidant activity of aqueous extract in rats	[144]
*Thymus* spp.	Epigeal part (Whole) (infusion)	Med	Circulatory system disorders	Blood depurative	Hepatoprotective and antioxidant activity of aqueous extract in rats	[144]
	Flowers/inflorescences/flowering tops (infusion)	Med	Respiratory tract infections	Balsamic Cough Colds and flu symptoms Expectorant, decongestant, emollient	Antimicrobial activity of aqueous extract	[170,199]
	Leaves (infusion)					
	Epigeal part (Whole) (infusion)					
*Laurus nobilis* L.	Fruits/infructescences/accessory fruits (infusion)	Med	Digestive tract disorders	Antiacid, gastritis, acid reflux Carminative Abdominal pain	Decoction in water of fruits with gastroprotective activity	[90]
*Linum usitatissimum* L.	Seeds (boiled or left in water overnight, then drunk)	Med	Digestive tract disorders	Laxative, intestinal motility	Maceration in how water to extract mucilages	[95]
*Malva sylvestris* L.	Leaves (infusion, compresses/baths with infusion)	Med	-	Analgesic	Aqueous extract with analgesic activity in rats (intraperitoneal administration)	[100]
	Leaves (infusion)	Med	Urinary tract disorders	Depurative	Nephroprotecive activity of aqueous extract of leaves and flowers	[106]
*Epilobium* spp.	Leaves (infusion)	Med	General condition	Anti-inflammatory	Aqueous extracts with activity on COX-1 and COX-2	[58]
					Aqueous extracts with anti-inflammatory activity in vitro	[59]
*Chelidonium majus* L.	Latex or sap (applied raw)	Med	Skin diseases and traumas	Skin tags and warts	Case report: 4-years child with warts treated with raw latex	[55]
*Cetraria islandica* (L.) Ach. subsp. *islandica*	Epigeal part (Whole) (infusion)	Med	Respiratory tract infections	Expectorant, decongestant, emollientSorethroat and hoarseness	Anti-inflammatory activity of aqueous extract in vitro	[51]
*Passiflora* spp.	Epigeal part (Whole) (infusion)	Med	Nervous system disorders	Promotes sleep	Effect of infusion on Subjective sleep quality	[126]
*Plantago major* L.	Leaves (washing with infusion)	Med	Oropharyngeal cavity affections	Gingivitis, toothache, mouth ulcers and abscesses	Anti-inflammatory activity of extract in deionized water in vivo	[129]
*Rosa canina* L.	Fruits/infructescences/accessory fruits (infusion)	Med	Respiratory tract infections	Decongestant Sorethroat	Different aqueous extracts of rose hips with anti-inflammatory activity	[140]
*Urtica dioica* L.	Leaves (applied as a poultice)	Med	Musculoskeletal system disorders and traumas	Rheumatism, Contusions, Hematoma	Leaves applied on thumb against osteoarthritis showed anti-inflammatory activity	[176]

## Supplementary Materials

The following are available online. Table S1. Bibliographic research on plants used for human and animal healthcare.
