# Supplementary material for: Using Medicinal Plants in Valmalenco (Italian Alps): From Tradition to Scientific Approaches
Source: Molecules. 2020 Sep 10;25(18):4144. doi: 10.3390/molecules25184144 (PMC7570945; doi:10.3390/molecules25184144)
Supplement: Supplementary file 1 [file molecules-25-04144-s001.pdf]

molecules

MDPI

Article

Using Medicinal Plants in Valmalenco (Italian Alps): from Tradition to Scientific Approaches

Martina Bottoni [1,2], Fabrizia Milani [1,2], Lorenzo Colombo [1,2], Kevin Nallio [1,2], Paola Sira Colombo [1,2], Claudia Giuliani [1,2], Piero Bruschi [3] and Gelsomina Fico [1,2,*]

[1] Department of Pharmaceutical Science, University of Milan, Milan 20133, Italy; martina.bottoni@unimi.it (M.B.); fabrizia.milani@unimi.it (F.M.); lorecolo.93@gmail.com (L.C.); kevin.nallio@studenti.unimi.it (K.N.); pasico19@virgilio.it (P.S.C.); claudia.giuliani@unimi.it (C.G.)

[2] Botanical Garden G.E. Ghirardi, Department of Pharmaceutical Science, University of Milan, Toscolano Maderno, Brescia 25088, Italy

[3] Department of Agricultural, Environmental, Food and Forestry Science and Technology, University of Florence, Florence 50144 code, Italy; piero.bruschi@unifi.it

* Correspondence: gelsomina.fico@unimi.it

Received: 7 August 2020; Accepted: 4 September 2020; Published: date

Abstract: This ethnobotanical survey was carried out in Caspoggio (Valmalenco, SO, Italy) with the purpose of investigating the traditional uses of medicinal plants. Moreover, a bibliographic research meant to validate or refute the uses, focusing on the potentially responsible compounds, was performed. Fifty-nine species, attributable to 30 families (Asteraceae, Pinaceae, Malvaceae, and Lamiaceae the most cited), were mentioned. *Arnica montana*, anti-inflammatory for traumas and musculoskeletal pains; *Pinus mugo*, expectorant; *Malva sylvestris*, anti-inflammatory and soothing; *Achillea moschata*, digestive. The compounds, responsible for the therapeutic activities, are often polyphenols and terpenoids: helenanin in *A. montana*, α-pinene, δ-3-carene, and limonene in *P. mugo*, gossypin and malvin in *M. sylvestris*, luteolin and apigenin in *A. moschata*. Scientific evidence for at least one of the traditional activities described was found for 50 species but only in 26 out of 196 works consulted, it is possible to make a comparison between investigated extracts and traditional preparations. This study is thus a stimulus to new phytochemical investigations, mimicking as much as possible the traditional preparations. This work is part of the European Interreg Italy-Switzerland B-ICE project, aimed at creating a management model for the ongoing climate change and searching for new sources of territory valorization as attractions for tourists.

Keywords: Valmalenco; ethnobotany; traditional uses; medicinal plants; climate change

Table S1. Bibliographic research on plants used for human and animal healthcare. For the complete Reference list, please see the main text.

Latin name Vernacular name	Traditional uses in Valmalenco				Bibliographic reference: Ethnobotany	Bibliographic reference: Biological activity	Bibliographic reference: Active compounds
	Part of the plant and preparation	Field of use	Category of use	Detailed use			
**Adoxaceae**							
Sambucus nigra L. Sambuco nero Sembüc	Fruits/infructescences/accessory fruits (eaten raw)	Med	Digestive tract disorders	Laxative, intestinal motility	[3,15,153]	[153]	Anthocyanins (Cyanidin-3-sambubioside, Chrysanthemin, Cyanidin-3,5-diglucoside); Proanthocyanidins (epicatechin); Flavonoids (Rutin, isoquercetin); Peptic polysaccharides; Lectins (SNA-IV, SNA-V) of berries extracts: immunostimulant, antiviral (against flu), and antimicrobic activities [155]
	Flowers/inflorescences/flowering tops (infusion)	Med	Gynecological disorders, obstetric and puerperal problems	Galactagogue			
	Flowers/inflorescences/flowering tops (infusion in water or milk, syrup)<br>Fruits/infructescences/accessory fruits (eaten raw)	Med	Respiratory tract infections	Bronchitis<br>Asthmatic bronchitis<br>Expectorant, decongestant, emollient<br>Sorethroat and hoarseness<br>Cough	[3,13,14,15,19,155,204,205,206]	[154,155,156]	
**Apiaceae**							
Foeniculum vulgare Mill. Finocchio selvatico, aneto	Epigeal part (Whole) (infusion)	Med	Digestive tract disorders	Gastric antispasmodic<br>Digestive<br>Carminative<br>Stomachache	WHO Monograph - Fructus Foeniculi [29] [69,206,207]	WHO Monograph - Fructus Foeniculi [29] [31,69,70]	E.O.: antispasmodic, carminative, anticolitic [69,70]
**Asteraceae**							
Achillea millefolium L. Achillea millefoglio, danedun	Epigeal part (Whole) (compresses with infusion)	Med	Skin diseases and traumas	Anti-inflammatory, disinfectant and wound healing, Emollient, soothing	OMS Monograph - Herba Millefolii [23]	[25] (Anti-inflammatory, antibacterial)	Sesquiterpene santamarin: anti-inflammatory (OMS

				[3,13,15,18,25]	[24] (Antibacterial)	Monograph - Herba Millefolii [23])
Flowers/inflorescences/flowering tops (infusion)	Med	Circulatory system disorders	Blood depurative	[3,25]		Dicaffeoylquinic acids, flavonoids, proazulenic sesquiterpene lactone (matricin): anti-inflammatory activity; E.O. antibacterial activity; flavonoids (apigenina, luteolin): estrogenic activity [25] E.O. (terpinolene, 1,8-cineol, thujone, γ-terpinene, borneol, camphor), rutin: antibacterial, antioxidant, and antinociceptive; apigenin 7-O-glucoside, luteolin 7-O-glucoside: anti-inflammatory; quercetin, apigenin, and luteolin antispasmodic; luteolin-7-O-beta-D-glucuronide: coleretic; dihydrodehydrodiconiferyl alcohol, and 9-O-beta-D-glucopyranoside: estrogenic [24]
Flowers/inflorescences/flowering tops (infusion) Leaves (infusion)	Med	Digestive tract disorders	Digestive Abdominal pain Stomachache Astringent, antidiarrheal	[3,13,15,19,24,25]	WHO Monograph - Herba Millefolii [23] [19,24,25]	
Epigeal part (Whole) (compress or bath with infusion) Leaves (compress or bath with infusion)	Med	Musculoskeletal system disorders and traumas	Contusions Sprains and dislocations Articolar pain and Inflammations		WHO Monograph - Herba Millefolii (Anti-inflammatory) [23] [25](Anti-inflammatory) [24]	
Flowers/inflorescences/flowering tops (infusion) Epigeal part (Whole) (wash or bath with infusion)	Med	Gynecological disorders, obstetric and puerperal problems	Anti-inflammatory and soothing Rash Menstrual pains Menopausal disorders	[3,13]	WHO Monograph - Herba Millefolii (Anti-inflammatory) [23] [24,25]	
Flowers/inflorescences/flowering tops (fumigation)	Med	Respiratory tract infections	Expectorant, decongestant, emollient	[15,24,25]	WHO Monograph - Herba Millefolii [23]	
Flowers/inflorescences/flowering	Med	General condition	Infections Relaxant footbath		[24,25]	

	tops (infusion, footbath)						
	Flowers/inflorescences/flowering tops (wash with infusion)	Vet	Cattle	Disinfectant for cattle legs wounds Intestinal disorders	[15]	WHO Monograph - Herba Millefolii [23]	
	Flowers/inflorescences/flowering tops (infusion)	Vet	Cattle Dogs	Intestinal disorders		WHO Monograph - Herba Millefolii [23]	
Achillea moschata Wulfen Erba iva, daneda	Epigeal part (Whole) (compresses with infusion)	Med	Skin diseases and traumas	Anti-inflammatory, disinfectant and wound healing	[3,26]	Antimicrobial [26]	Phenolic and caffeic compounds (7-O-glucosyl luteolin, 7-O-β-glucosyl apigenin, 3-O-β-glucosyl-kaempferol, Luteolin, Apigenin, 3-O-β-Rutinosyl-isorhamnetin, 3-O-β -Glucosyl-isorhamnetin 5-O-caffeoylquinic acid, 4,5-dicaffeoylquinic acid): antioxidant activity; E.O. (camphor, 1-8-cineole, bornylacetate): antibacterial [26]
	Flowers/inflorescences/flowering tops (infusion)	Med	Circulatory system disorders	Hypotensive	[13,26]		
	Flowers/inflorescences/flowering tops (infusion) Epigeal part (Whole) (infusion)	Med	Digestive tract disorders	Gastric antispasmodic Digestive Carminative Abdominal pain Stomachache	[3,13,14,15,22,26,208,209]		
	Epigeal part (Whole) (footbath/bath with infusion)	Med	Musculoskeletal system disorders and traumas	Inflammations and pain	[13]		
	Flowers/inflorescences/flowering tops (infusion)	Med	Gynecological disorders, obstetric and puerperal problems	Menstrual pains	[13,15,26]		

	Epigeal part (Whole) (infusion)	Med	Nervous system disorders	Sedative hypnotic, promotes sleep	[3,15,26]		
	Epigeal part (Whole) (infusion)	Med	Respiratory tract infections	Cough	[3,15,26]		
	Flowers/inflorescences/flowering tops (infusion)	Med	General condition	Anti-inflammatory	[3,15,22,26]		
	Flowers/inflorescences/flowering tops (infusion)	Vet	Cattle	Intestinal problems, bloating	[2,3,13,15,26]		
	Underground organs (roots/bulbs/tubers/rhizomes) (decoction)	Med	Urinary tract disorders	Remineralizing Urinary tract depurative			Helenanin (sesquiterpene lactone): analgesic and anti-inflammatory (WHO Monograph - Flos Arnicae [29] [30,33,34]<br><br>Helenanin (sesquiterpene lactone): atimicrobial [31]<br><br>MeOH extract *per os* (quinic acids, inositol esters, rutin, gallic acid): antinflammatory [32]<br><br>Helenanin and dihydro-helenanin: anti-inflammatory [30,31,36]<br><br>Flavonoids and phenols: antioxidant [30]
Arnica montana L. subsp. *montana* Arnica	Flowers/inflorescences/flowering tops (exudate/EtOH extract application)<br><br>Leaves (boiled and applied)	Med	Skin diseases and traumas	Anti-inflammatory, disinfectant and wound healing	[2,13,14,15,22,30]	WHO Monograph - Flos Arnicae [29] [30,36]	
	Flowers/inflorescences/flowering tops (exudate/exudate in ointment or compresses with infusion)	Med	Musculoskeletal system disorders and traumas	Contusions Articolar pain and inflammations Rheumatic pain Muscle inflammations and pain	WHO Monograph - Flos Arnicae [29] [2,3,13,14,15,30]	WHO Monograph - Flos Arnicae [29] [30,31,32,36]	
	Flowers/inflorescences/flowering tops	Med	Digestive tract disorders	Stomachache Stomach depurative	WHO Monograph -		

	(infusion)				Flos Arnicae [29]		
	Flowers/inflorescences/flowering tops (exudate application)	Med	General condition	Anti-inflammatory Analgesic Infections	[15,30]	WHO Monograph - Flos Arnicae [29] [30,31,33,34,35,36]	
Artemisia absinthium L. Assenzio	Flowers/inflorescences/flowering tops (infusion)	Med	Digestive tract disorders	Digestive	[2,3,13,14,15,22,205]	[31,37,38] (Opposite effect - [40])	Bitter taste due to sesquiterpenes lactones (absinthin, anabsinthin, arthabsin, anabsin): digestive [31,37,39]
	Flowers/inflorescences/flowering tops (infusion)	Med	Nervous system disorders	Cephalea due to digestive disorders	[14]		
	Epigeal part (Whole) (eaten raw)	Vet	Cattle	Promotes digestion	[13,14,15]	[31,37]	
Artemisia genipi Weber Genepì	Epigeal part (Whole) (infusion)	Med	Digestive tract disorders	Digestive	[2,3,13,15,18]	[39,41]	Some of the sesquiterpenes lactones (such as santamarine) of génépi may activate hTAS2R46 bitter taste receptors [39,41]
	Epigeal part (Whole) (infusion)	Med	Gynecological disorders, obstetric and puerperal problems	Menstrual pains			
	Epigeal part (Whole) (infusion)	Med	Respiratory tract infections	Colds	[15,18]	[39,41]	
Calendula officinalis L. Calendula	Flowers/inflorescences/flowering tops (compresses with infusion, ointment)	Med	Skin diseases and traumas	Anti-inflammatory, disinfectant and wound healing Sunburns, burns, frostbites, redness and rash	WHO Monograph - Flos Calendulae [43] [3,13,14,15,44,47,206]	WHO Monograph - Flos Calendulae [43] [31,44,45,46,47]	Flavonoids (quercitin): antioxidant [45] Triterpenoid esters (faradiol-3-O-laurate, faradiol-3-O-palmitate e

				Emollient, soothing<br>Insect bites			faradiol-3-O-myristate): anti-inflammatory.<br>Quercitin: partially inhibits collagen degradation and MMP2 activity.<br>Quercetin-3-O-glucoside and rutin stimulate primary fibroblasts.<br>[44]
	Epigeal part (Whole) (compresses with infusion)	Med	Circulatory system disorders	Promotes circulation	WHO Monograph - Flos Calendulae [43]<br>[2,15]	WHO Monograph - Flos Calendulae [43]<br>[46,48]	
	Flowers/inflorescences/flowering tops (infusion)	Med	Urinary tract disorders	Cystitis and other inflammation of the urinary tract			Carotenoids or their degradation compounds may promote wound healing more than triterpenes<br>[47]
	Flowers/inflorescences/flowering tops (macerated oil, ointment)	Med	Musculoskeletal system disorders and traumas	Contusions<br>Articolar pain and inflammations	WHO Monograph - Flos Calendulae [43]<br>[13,15]	[210]	Terpenoids and flavonoids: anti-inflammatory and antiedema<br>[46]
	Flowers/inflorescences/flowering tops	Vet	Alpacas	Animal skin allergic reactions			
Carduus nutans L.<br>Cardo svizzero	Flowers/inflorescences/flowering tops (infusion)	Med	Urinary tract disorders	Depurative	[211,212]		Synergic activity of different non-polar compounds (i.e. palmitic, oleic, and linoleic acids): antiviral and anti-inflammatory [49]
	Flowers/inflorescences/flowering tops (eaten raw)	Vet	Cattle	Galactagogue			
	Flowers/inflorescences/flowering tops (eaten raw)	Vet	Cattle	Throat depurative		[49]	Flavonoids luteolin and apigenin: anti-inflammatory and antioxidant [211,212]

Carlina acaulis L. subsp. *intybus*<br>Carlina bianca, Pane degli alpini	Flowers/inflorescences/flowering tops (eaten raw)	Vet	Cattle	Galactagogue			
	Flowers/inflorescences/flowering tops (eaten raw)	Vet	Cattle	Throat depurative			
Cichorium intybus L.<br>Catalogna	Leaves (infusion)	Med	Urinary tract disorders	Depurative	[3,15,56,57,206,213,214]	[56,57]	
Matricaria chamomilla L.<br>Camomilla	Flowers/inflorescences/flowering tops (compresses with infusion)	Med	Ophthalmic ailments	Ocular anti-inflammatory	WHO Monograph – Flos Chamomillae [43]<br>[3,14,15,22,206,215]	[31,67]	Apigenin: anti-inflammatory [31,108,109]; ansiolitic and antispasmodic [31,108]<br><br>Bisabolol: antispasmodic [108]<br><br>Bisabolol, chamazulene: antibacterial and antifungal [31]; Anti-inflammatory (WHO Monograph – Flos Chamomillae [43]; [31])<br><br>Matricin: anti-inflammatory and spasmolytic (WHO Monograph – Flos Chamomillae [43])
	Flowers/inflorescences/flowering tops (infusion)	Med	Digestive tract disorders	Abdominal pain<br>Stomachache<br>Gastric antispasmodic	[3,15,67,109,111,205,206]	WHO Monograph – Flos Chamomillae [43]<br>[31,108,109,110]	
	Flowers/inflorescences/flowering tops (infusion)	Med	Nervous system disorders	Sedative hypnotic, promotes sleep	[3,13,14,15,31,109,111,112,205,206]	WHO Monograph – Flos Chamomillae [43]<br>[31,108,109,111,112]	
	Flowers/inflorescences/flowering tops (bath/washing with infusion)	Med	General condition	Emollient, soothing	[15]	WHO Monograph – Flos Chamomillae [43]<br>[31]	Flavonoids (apigenina and luteolin): anti-inflammatory (WHO Monograph – Flos Chamomillae [43]; ansiolitic [111]

							Apigenin-7-O-Glucoside found in aqueos extracts [216]
	Flowers/inflorescences/flowering tops (compresses with infusion)	Vet	All	Ocular anti-inflammatory, conjunctivitis	[168]	[31,67]	
Silybum marianum (L.) Gaertn. Cardo mariano	Flowers/inflorescences/flowering tops (infusion)	Med	Circulatory system disorders	Blood depurative		[157]	Silymarin and silybin: anti hepatotoxic (WHO Monograph - Fructus Silybi Mariae [23]; [31,157,158]); anti-inflammatory and antioxidant. (WHO Monograph - Fructus Silybi Mariae [23]; [31]) Silybin detected in inflorescences [217]
	Flowers/inflorescences/flowering tops (infusion)	Med	Digestive tract disorders	Liver depurative	[22,157]	WHO Monograph – Fructus Silybi Mariae [23] [31,157,158]	
Taraxacum spp. Tarassaco Zicòria/dent de can	Flowers/inflorescences/flowering tops (syrup)	Med	Oropharyngeal cavity affections	Sorethroat		[163] (Anti-inflammatory) [162]	Bitter Sesquiterpene glucosides 14-O-beta-D-glucosyl-11,13-dihydro-taraxinic acid and 14-O-beta-D-glucosyl-taraxinic acid (from MeOH extract): anti-inflammatory [166,218] Triterpene uvaol (from dried flowers): anti-inflammatory [168]
	Flowers/inflorescences/flowering tops (applied raw) Latex or sap (latex raw) Epigeal part (Whole) (compresses with infusion)	Med	Skin diseases and traumas	Acne, pimples, foruncles, skin abscesses Anti-inflammatory, disinfectant and wound healing Insect bites	[15,219]	[163] (Anti-inflammatory)	

Underground organs (roots/bulbs/tubers/rhizomes) (eaten)	Med	Circulatory system disorders	Blood depurative	[2,19,165]		Sesquiterpene lactones and phenylpropanoids: anti-inflammatory. Terpenoids and bitter lactones: could be active on the liver [163]
Flowers/inflorescences/flowering tops (macerated in alcohol, eaten raw sometimes with leaves, infusion)<br><br>Epigeal part (Whole) (syrup)	Med	Digestive tract disorders	Gastric antispasmodic<br>Digestive<br>Liver depurative	[13,14,15,22,164,165,205,206,220]	[163,164,165]	
Flowers/inflorescences/flowering tops (infusion)<br><br>Leaves (infusion or decoction)<br><br>Epigeal part (Whole) (infusion)<br><br>Underground organs (roots/bulbs/tubers/rhizomes) (infusion or decoction)	Med	Urinary tract disorders	Kidney stones<br>Cystitis and other inflammation of the urinary tract<br>Urinary tract depurative<br>Diuretic	[2,13,19,163,165,206,219]	[163,165,166,167]	
Flowers/inflorescences/flowering tops (syrup, infusion)	Med	Respiratory tract infections	Expectorant, decongestant, emollient<br>Flu and flu syndromes<br>Sorethroat and hoarseness<br>Cough	[13,15,163,165]		

	Flowers/inflorescences/flowering tops (syrup)	Med	General condition	Anti-inflammatory	[3,15,163,165]	[163,165,168]	
	Flowers/inflorescences/flowering tops (applied raw)	Vet	Other animals	Anti-inflammatory for infected wounds	[219]	[163,168] (Anti-inflammatory)	
				**Betulaceae**			
Betula pendula Roth<br>Pendula	Leaves (infusion)	Med	Circulatory system disorders	Hypocholesterolemic	[42]		Acid betulinic: anti-inflammatory and diuretic [42]<br><br>Poliphenols (catechins, p-coumaric acid, myricetin, quercetin, and kaempferol): antioxidant [221]
	Leaves (infusion)	Med	Urinary tract disorders	Urinary tract depurative<br>Diuretic	[3,15,42,221]	[42]	
	Leaves (infusion)<br><br>Latex or sap (drunk raw)	Med	General condition	Tonic and corroborant	[42]		
				**Brassicaceae**			
Brassica oleracea L.<br>Verza	Leaves (applied as a poultice)	Med	Musculoskeletal system disorders and traumas	Arthrosis<br>Contusions<br>Sprains and dislocations<br>Rheumatic pain<br>Inflammations and pain<br>Articolar pain and inflammations	[3,205,206]		
	Leaves (applied as a poultice)	Med	Respiratory tract infections	Improves breathing			
				**Caprifoliaceae**			
Valeriana officinalis L.<br>Valeriana	Epigeal part (Whole) (infusion)	Med	Nervous system disorders	Sedative hypnotic, promotes sleep	[15,195,206]	[189,190,191,192,193,194,195] ([196] no statistically significant	Valepotriates, valerenic acid and derivatives, E.O.: sedative [222]<br>Valerenic acid and E.O. [194]

						difference to placebo)	Sesquiterpenes, valerenic acid [168,195]
**Cupressaceae**							
Juniperus communis L.<br>Ginepro<br>Pedüli de càvar	Fruits/infructescences/accessory fruits (infusion)<br><br>Epigeal part (Whole) (infusion)	Med	Digestive tract disorders	Digestive	[2,3,14,15,18,19,77,78,223,224]	[77,78,79]	E.O.: digestive [79]<br><br>E.O. (α-pinene 20-50%, sabinene <20%, β-pinene 1-12%, β-myrcene 1-30%) [77,78]
	Fruits/infructescences/accessory fruits (infusion)	Med	Nervous system disorders	Sedative hypnotic, promotes sleep	[225]		
	Fruits/infructescences/accessory fruits (infusion, salve/ointment)	Med	Respiratory tract infections	Colds<br>Expectorant, decongestant, emollient	[3,13,14,15,78,224]		
**Equisetaceae**							
Equisetum arvense L.<br>Erba cavallina, equiseto, coda cavallina	Herbaceous stems/scapes (infusion)	Med	Urinary tract disorders	Diuretic<br>Depurative<br>Kidney stones<br>Remineralizing	[2,3,13,14,15,62]	[60,61,62]	High concentrations of flavonoids, phenolic compound, and mineral salts: mild diuretic action; silicon salts: may indicate remineralisation properties [62]<br><br>Kynurenic acid: anti-inflammatory and analgesic [64,66]<br><br>B-sitosterol, campesterol, isofucosterol: anti-inflammatory steroidal effects
	Herbaceous stems/scapes (applied as a poultice)	Med	Musculoskeletal system disorders and traumas	Contusions	[3]	[60,61,63,64,65,66]	
	Herbaceous stems/scapes (infusion)	Med	Gynecological disorders, obstetric and puerperal problems	Menstrual pains			

	Herbaceous stems/scapes (infusion)	Med	General condition	Analgesic		[61,63,64,65,66]	[65]
			**Ericaceae**				
Vaccinium myrtillus L. Mirtillo nero	Fruits/infructescences/accessory fruits (eaten raw)	Med	Ophthalmic ailments	Promotes eyesight and eyes health	WHO Monograph - Fructus Myrtilli [146] [3,13,14,15,187] (refuted by [185])	WHO Monograph - Fructus Myrtilli [146] [177,178,179,180,181,182,183,184]	Phenolic compounds (in particular anthocyanins): improve ocular microcirculation (WHO Monograph - Fructus Myrtilli) [146], [183]); therapeutic properties (nonspecific) [187]<br><br>Cyanidin, delphinidin, and malvidin: protect from retinal damage [179,181]
	Fruits/infructescences/accessory fruits (eaten raw)	Med	Circulatory system disorders	Promotes circulation	WHO Monograph Fructus Myrtilli [146] [3,13,15,187]	WHO Monograph - Fructus Myrtilli [146] [178,183,186]	
	Fruits/infructescences/accessory fruits (eaten raw)	Med	Urinary tract disorders	Promotes urinary tract health	WHO Monograph - Fructus Myrtilli [146] [3,187,204]	[187]	
Vaccinium vitis-idaea L. Mirtillo rosso Gaüdì	Fruits/infructescences/accessory fruits (eaten raw)	Med	Ophthalmic ailments	Promotes eyesight and eyes health		[180,181]	Compounds active on urinary tract: high molecular weight proanthocyanidins (condensed tannins) [183]; phenolic compounds [226]; arbutin and derivatives, tannins [187]
	Fruits/infructescences/accessory fruits (eaten raw)	Med	Circulatory system disorders	Circulation, veins and blood vessels			
	Fruits/infructescences/accessory fruits (eaten raw)	Med	Urinary tract disorders	Promotes urinary tract health	[3,13,187,204,226]	[183,187,188]	
			**Gentianaceae**				
Gentiana lutea L. subsp. *lutea* Riszanzana	Underground organs (roots/bulbs/tuber	Med	Circulatory system disorders	Blood depurative			Bitter monoterpenes (gentiopicroside, swertiamarin, sweroside e

	s/rhizomes) (decoction)						amarogentin): digestive and eupeptic WHO Monograph - Radix Gentianae Scabrae [29]) Gentiopicroside, amarogentin: gastroprotective [71,227]
	Underground organs (roots/bulbs/tubers/rhizomes) (decoction, grappa)	Med	Digestive tract disorders	Digestive Stomach anti-inflammatory Liver anti-inflammatory Stomachache Vermifuge	WHO Monograph - Radix Gentianae Luinfusione [29] [2,14,15,22,227]	WHO Monograph - Radix Gentianae Luinfusione [29] [31,37,71]	
	Underground organs (roots/bulbs/tubers/rhizomes) (decoction)	Med	Urinary tract disorders	Urinary tract depurative	[15,227]		
	Underground organs (roots/bulbs/tubers/rhizomes) (macerated oil)	Med	Musculoskeletal system disorders and traumas	Contusions Inflammations and pain			
	Flowers/inflorescences/flowering tops (decoction)	Med	General condition	Immunomodulant Tonic and corroborant	WHO Monograph - Radix Gentianae Luinfusione [29] [15]		
	Underground organs (roots/bulbs/tubers/rhizomes) (decoction)	Vet	Cattle	Carminative			
				**Hypericaceae**			
Hypericum perforatum L. Iperico	Flowers/inflorescences/flowering tops (macerated oil)	Med	Skin diseases and traumas	Sunburns, burns, frostbites, redness and rash Anti-inflammatory, disinfectant and	WHO Monograph – Herba Hyperici [23] Guarrera 2005	WHO Monograph – Herba Hyperici [23] [31,72]	Hyperforin, hypericin, and pseudohypericin: antimicrobial; hyperforin and flavonoids: antioxidant and anti-

				wound healing<br>Psoriasis<br>Insect bites	[2,3,13,14,15,<br>19,22,72,205,<br>206]		inflammatory<br>[72,73]<br>hyperforin and hypericin:<br>antidepressant<br>(WHO Monograph - Herba Hyperici [23], [31])
	Epigeal part (Whole) (macerated oil rubbed)	Med	Digestive tract disorders	Abdominal pain			
	Epigeal part (Whole) (infusion)	Med	Urinary tract disorders	Urinary tract depurative	WHO Monograph – Herba Hyperici [23]<br>[72]		
	Flowers/inflorescences/flowering tops (macerated oil, compresses with infusion)	Med	Musculoskeletal system disorders and traumas	Contusions<br>Sprains and dislocations<br>Articolar pain and inflammations<br>Muscle inflammations and pain	[2,3,13,14,15,<br>22,72]	[72,73]	
	Epigeal part (Whole) (macerated oil, infusion)	Med	Nervous system disorders	Lumbago and neuralgia<br>Antidepressant<br>Sedative hypnotic, promotes sleep	WHO Monograph – Herba Hyperici [23]<br>[3,13,15,72]	WHO Monograph – Herba Hyperici [23]<br>[31,73,74,75,76]	
	Epigeal part (Whole) (macerated oil)	Med	General condition	Lymphatic system anti-inflammatory			
**Iridaceae**							
Crocus vernus (L.) Hill<br>Croco, bucaneve	Epigeal part (Whole) (eaten raw)	Vet	Cats	Observed a thrill-like effect when ingested			
**Lamiaceae**							
Lamium album L.<br>Ortica bianca	Leaves (infusion)	Med	Urinary tract disorders	Diuretic	[13,80]	[80,81]	

	Flowers/inflorescences/flowering tops (macerated oil)	Med	Afflictions of the ear	Otitis	[3]		
Lavandula angustifolia Mill. Lavanda	Leaves (compresses with infusion)	Med	Skin diseases and traumas	Anti-inflammatory, disinfectant and wound healing	WHO Monograph – Flos and Aetheroleum Lavandulae [29] [15]	WHO Monograph - Aetheroleum Lavandulae [29] [82,84,85]	E.O.: anti-inflammatory (WHO Monograph - Flos Lavandulae [29]); wound healing ([228], [229]- Flowers, [82]- Leaves); sedative (WHO Monograph - Flos Lavandulae [29]; particularly linalool [31])
	Flowers/inflorescences/flowering tops (infusion)	Med	Nervous system disorders	Sedative hypnotic, promotes sleep	[3,15,206]	WHO Monograph - Flos Lavandulae [29] [31,86]	
	Flowers/inflorescences/flowering tops (salve/ointment)	Med	General condition	Anti-inflammatory		[83,84]	
Melissa officinalis L. Melissa	Flowers/inflorescences/flowering tops (applied as a poultice)	Med	Gynecological disorders, obstetric and puerperal problems	Menstrual pains	WHO Monograph - Folium Melissae [23] [230]	[113,114]	
Mentha longifolia (L.) Huds. Menta	Leaves (infusion)	Med	General condition	Tonic and corroborant			
Mentha x piperita L. Menta piperita	Leaves (gargles/washings with infusion)	Med	General condition	Anti-inflammatory	[115]	[115,116,117]	E.O. (menthol 30,69%, menthone 14,51%, and menthyl acetate 12,86%): anti-inflammatory [116]
Nepeta cataria L. Erba gatta	Epigeal part (Whole) (eaten raw)	Vet	Cats	Given to the kittens by the mother			

	Epigeal part (Whole) (infusion)	Med	Digestive tract disorders	Promotes digestion	[2,15,121,206,231]		Carvacrol: anti-inflammatory [118,119]
Origanum vulgare L. Origano	Epigeal part (Whole) (compresses with infusion)	Med	Ophthalmic ailments	Conjunctivitis		[118,119,120,121]	High carvacrol E.O.: anti-inflammatory; E.O.: antimicrobial against *Pseudomonas aeruginosa, Staphylococcus aureus,* and *Escherichia coli* (often found in ocular infections bacterial profiles [232]) [120]
Rosmarinus officinalis L. Rosmarino	Epigeal part (Whole) (infusion)	Med	Digestive tract disorders	Liver depurative	[141]	WHO Monograph Aetheroleum Rosmarini and Folium Rosmarini [146]<br><br>[141,142,143,144,145]	E.O. (α-pinene 18–26%, camphene 8–12%, β-pinene 2.0–6.0%, β-myrcene 1.5–5.0%, limonene 2.5–5.0%): (WHO Monograph Aetheroleum Rosmarini and Folium Rosmarini [146])<br><br>E.O. (1,8-cineole 43.77%, camphor 12.53%, and α-pinene 11.51%): antioxidant and hepatoprotective [142]<br><br>In the aqueous extract with antioxidant and hepatoprotective properties catechin, coumarin, cinnamic acid, and rutin are detected [144]
Salvia officinalis L. Salvia	Leaves (applied raw)	Med	Oropharyngeal	Gingivitis	[2,3,14,15,150,207,231]	[149,150]	Flavonoidic fraction, rosmarinic acid, manool,

			cavity affections				carnosol, and ursolic acid: anti-inflammatory [152]<br><br>E.O. (1,8-cineol, thujone, and camphor), non-volatile compounds in MeOH extract: antimicrobial and antifungal (i.e. for oral cavity affections) and anti-inflammatory ([150,231])<br><br>Ursolic acid and other diterpenes: anti-inflammatory [150]<br><br>MeOH extract with high contents of rosmarinic, ursolic, caffeic, and oleanolic acids; borneol: anti-inflammatory [151]
	Leaves (infusion)	Med	Digestive tract disorders	Depurative and abdominal bloating	[150,151,152]	[150]	
	Leaves (infusion)	Med	Urinary tract disorders	Depurative	[15]		
	Leaves (compresses with infusion)	Med	Musculoskeletal system disorders and traumas	Anti-inflammatory for pains and sprains	[152,231]	[152]	
	Leaves (infusion)	Med	Respiratory tract infections	Colds	[3,15,150,207,231]		
	Leaves (washings with infusion)	Med	General condition	Anti-inflammatory	[150,231]	[150,151,152]	
Thymus spp. Timo	Epigeal part (Whole) (infusion)	Med	Other	Depurative			Phenolic compounds, carvacrol, thymol, and others (high variability): antibacterial and antioxidant [170,234,202]<br><br>E.O. and thymol: antibacterial [171]
	Epigeal part (Whole) (infusion)	Med	Circulatory system disorders	Blood depurative	[199,204]	[144]	
	Flowers/inflorescences/flowering tops (infusion)<br><br>Epigeal part (Whole) (infusion)	Med	Digestive tract disorders	Emollient, stomachache	[170,171,204,207,199]		

	Epigeal part (Whole) (infusion)	Med	Urinary tract disorders	Diuretic	[15,18,170] ([199] traditionally used as anti-diuretic)		
	Epigeal part (Whole) (infusion)	Med	Nervous system disorders	Relaxant	[3,170,171]		
	Flowers/inflorescences/flowering tops (infusion)<br><br>Leaves (infusion)<br><br>Epigeal part (Whole) (infusion)	Med	Respiratory tract infections	Balsamic<br>Cough<br>Colds and flu symptoms<br>Expectorant, decongestant, emollient	[2,13,14,15,18,19,170,171,199,204,206]	[169,170,171,172]	
	Epigeal part (Whole) (salve/ointment)	Med	General condition	Anti-inflammatory	[170]	([170] antioxidant) [171]	
**Lauraceae**							
Laurus nobilis L.<br>Alloro	Fruits/infructescences/accessory fruits (infusion)<br><br>Leaves (infusion)	Med	Digestive tract disorders	Antiacid, gastritis, acid reflux<br>Carminative<br>Abdominal pain	[3,15,31,89,90,206,235,236]	[89,90,91]	1.8 cineol and terpinene: gastroprotective activity [89,90]<br><br>E.O.: anti-inflammatory and analgesic [92,93]
	Leaves (infusion)	Med	Gynecological disorders, obstetric and puerperal problems	Menstrual pains	[92,93]	[92,93]	
**Linaceae**							
Linum usitatissimum L.<br>Lino	Seeds (boiled or left in water overnight, then drunk)	Med	Digestive tract disorders	Laxative, intestinal motility	[2,3,13,15,96]	[94,95,96]	Oil and mucilages: laxative [95]

	Epigeal part (Whole) (boiled and applied as a poultice)	Med	Respiratory tract infections	Expectorant, decongestant, emollient	[2,3,13,14,15,18,206]	[97]	
				**Malvaceae**			
Malva sylvestris L. Malva	Leaves (gargles with infusion)	Med	Oropharyngeal cavity affections	Oral cavity inflammation Toothache Gingivitis	[3,14,100,205,206,237,238]	[98,99,100,101]	Anti-inflammatory activity: flavonoids and mucilages [100,238]; polysaccharide of mucilaginous complex [237]; polyphenols [98]; Malvidin-3-glucoside [105] Phenolic compounds: protect kidney function [106]
	Leaves (compresses with infusion)	Med	Skin diseases and traumas	Anti-inflammatory Emollient Regenerative	[2,3,15,22,100,205,206,207,237,238]	[100,101,102,103,104,105]	
	Leaves (compresses with leaves)	Med	Ophthalmic ailments	Anti-inflammatory, Eye irritations	[3,100]	[100,101]	
	Leaves (bath/washing with infusion)	Med	Early infancy ailments	Soothing, anti-inflammatory for diaper rash	[14,100,237]	[100,101]	
	Leaves (infusion, washing with infusion)	Med	Other	Anti-inflammatory, refreshing, allergies, relaxant footbath	[3,100,237,238]	[100,101]	
	Leaves (infusion)	Med	Digestive tract disorders	Stomachache, Abdominal pain, Intestinal anti-inflammatory, promotes digestion	[2,14,15,22,100,205,237,238]		
	Leaves (infusion)	Med	Urinary tract disorders	Depurative	[2,15,100]	[106,107]	
	Leaves (compresses with infusion)	Med	Musculoskeletal system disorders and traumas	Anti-inflammatory for sprains, tired legs	[3,100]		

	Leaves (bath/washing with infusion)	Med	Gynecological disorders, obstetric and puerperal problems	Anti-inflammatory, facilitates childbirth	[3,15,100,206]	[101]	
	Leaves (bath)	Med	Nervous system disorders	Relaxant, sedative	[100]		
	Leaves (infusion)	Med	Respiratory tract infections	Colds	[15,100,206,238]		
	Leaves (compresses, Bath/washing with infusion)	Med	General condition	Anti-inflammatory, soothing	[3,100,205,237,238]	[100,101]	
Tilia spp. Tiglio	Flowers/inflorescences/flowering tops (infusion)	Med	Digestive tract disorders	Promotes digestion	[15,239,240,241]		
	Flowers/inflorescences/flowering tops (infusion)	Med	Respiratory tract infections	Cough	[2,13,14,15,22,204,206,239,240,241]		
**Oleaceae**							
Fraxinus excelsior L. Frassino, Frassino maggiore	Leaves (infusion)	Med	Digestive tract disorders	Laxative, intestinal motility			
	Leaves (infusion)	Med	Urinary tract disorders	Diuretic, Urinary tract depurative	[15,22]		
**Onagraceae**							
Epilobium sp. Epilobio	Leaves (infusion)	Med	Urinary tract disorders	Urinary tract depurative	[31,58,59]	[59]	Anti-inflammatory activity: myricetin-3-O-glucuronide [58,59]; oenothein B, myricetine-3-O-glucoside, myricetine-3-O-
	Leaves (infusion)	Med	General condition	Anti-inflammatory	[58,59]	[58,59]	

							galactoside, myricetine-3-O-rhamnoside and 3-O-glucuronides of quercetin e keampferol [58]<br><br>Antioxidant activity: phenols [58,59]
				**Orobanchaceae**			
Euphrasia rostkoviana Hayne<br>Eufrasia	Flowers/inflorescences/flowering tops (compresses with infusion)	Med	Ophthalmic ailments	Ocular anti-inflammatory	[3,13,15,68,242]	[31,67,68]	Flavonoids: antioxidant [67]; anti-inflammatory [67,68]<br><br>Polyphenols: anti-inflammatory [68]<br><br>Active substances of the *Euphrasia herb*: iridoglicozoids (aucubin), tannins, phenolic acids, etheric oils, resin substances and flavonoids [68]
				**Oxalidaceae**			
Oxalis acetosella L.<br>Cücülì, pan del cucù	Leaves (infusion)	Med	Digestive tract disorders	Digestive	[3,15]		
				**Papaveraceae**			
Chelidonium majus L.<br>Celidonia, Erba dei porri	Latex or sap (applied raw)	Med	Skin diseases and traumas	Skin tags and warts	[2,3,13,14,15,55,132,205,206]	[54,55]	Anti-inflammatory activity: alkaloids [55,132,243]in particular sanguinarine, chelidonine, chelerythrine, berberine e coptisine [243]
				**Parmeliaceae**			
Cetraria islandica (L.) Ach. subsp. *islandica*<br>Lichene<br>Nichel	Epigeal part (Whole) (infusion)	Med	Respiratory tract infections	Expectorant, decongestant, emollient<br>Sorethroat and hoarseness	[2,3,13,15,18,22,51]	[50,51,52]	Polysaccharides (lichenan and isolichenan especially active): antitussive [50]; immunomodulating [52];

	Epigeal part (Whole) (salve/ointment)	Med	General condition	Anti-inflammatory	[51]	[51,53]	(lichenan only) anti-inflammatory [51]
				**Passifloraceae**			
Passiflora spp.. Passiflora	Epigeal part (Whole) (infusion)	Med	Nervous system disorders	Promotes sleep	[124,125]	[122,123,124,125,126]	Flavonoids and perhaps GABA: ansiolitic [125]
				**Pinaceae**			
Larix decidua Mill. Larice	Resin (topical application)	Med	Oropharyngeal cavity affections	Gingivitis, toothache, mouth ulcers and abscesses			Procyanidins and larixinol: antioxidant [244]<br><br>Lignans and flavonols (wood-bark): anti-inflammatory [87,88], abietane, pimarane, and labdane type diterpenes: anti-inflammatory [87]
	Resin (ointment)	Med	Skin diseases and traumas	Anti-inflammatory, disinfectant and wound healing Sunburns, burns, frostbites, redness and rash	[2,3,14,244]	[31,87,88]	
	Resin (melted, mixed with red wine and applied to put in plaster)	Med	Musculoskeletal system disorders and traumas	Broken bones	[2,13,15]		
	Fruits/infructescences/accessory fruits (syrup)<br><br>Buds (syrup)<br><br>Resin (inalation)	Med	Respiratory tract infections	Expectorant, decongestant, emollient Cough	[2,3,13,14,19,87,244]	[88]	
	Resin (ointment)	Med	General condition	Anti-inflammatory	[244]	[31,87]	
Picea abies (L.) H.Karst Abete rosso	Resin (topical application)	Med	Oropharyngeal cavity affections	Gingivitis, toothache, mouth ulcers and abscesses	[15]	[88,131,132]	Antibacterial activity: bark flavonoids [88]; piperidine alkaloids (epidihydropinidine) [131]

	Resin (topical application)	Med	Skin diseases and traumas	Anti-inflammatory, disinfectant and wound healing	[3,132]		Antifungal activity: epidihydropinidine [131]<br>Antimicrobial: buds E.O. [132]
	Buds (syrup)	Med	Respiratory tract infections	Cough	[3,13,15,242]	[31,88]	Anti-inflammatory activity: bark stilbens and buds E.O. [132]; lignans and bark flavonols [88]<br><br>Activity antioxidant: bark flavonoids [88]<br><br>Mucolytic, hyperemic and antiseptic activities: E.O. [31]
Pinus cembra L. Pino cembro	Pine cones (syrup)	Med	Respiratory tract infections	Cough, Sorethroat	[2]	[133] Antimicrobial	Phenolics, flavonoids, and proanthocyanidins: antimicrobial activity [133]
Pinus mugo Turra Pino mugo, Pino montano	Resin (topical application, ointment)	Med	Skin diseases and traumas	Anti-inflammatory, disinfectant and wound healing Sunburns, burns, frostbites, redness and rash		[134]	E.O. needles, twigs and cones: anti-inflammatory. (Prevalence of α-pinene in needles, δ-3-carene in twigs and cones, limonene in twigs) [134]
	Resin (topical application)	Med	Other medicinal	Anti-inflammatory		[134]	
	Immature pine cones (infusion)	Med	Digestive tract disorders	Digestive	[3]		
	Resin (melted, filtered and used to put in plaster)	Med	Musculoskeletal system disorders	Sprains and dislocations Broken bones			

			and traumas				
	Buds (syrup)<br><br>Immature pine cones (syrup)	Med	Respiratory tract infections	Expectorant, decongestant, emollient<br>Sorethroat and hoarseness<br>Colds<br>Cough	[2,3,13,15,19]	[135,136]	
	Resin (topical application)	Med	General condition	Infections		[134]	
	Resin (topical application)	Vet	Cattle	Disinfectant and wound healing			
				**Plantaginaceae**			
Plantago major L.<br>Porceléra/ciantàna/plantàna	Leaves (washing with infusion)	Med	Oropharyngeal cavity affections	Gingivitis, toothache, mouth ulcers and abscesses		[127,128,129]	Therapeutic properties due to flavonoids, alkaloids, terpenoids, phenolic compounds (caffeic acid derivatives), iridoid glycosides, fatty acids, polysaccharides, and vitamins.<br>In particular, polyphenols (plantamajoside) and polysaccharides: wound healing activity;<br>Flavonoids (plantaginin, baicaleina, and hispidulin): antioxidant (may contribute to wound healing)<br>Iridoid glycosides (aucubin) and flavonoids (baicalein and hispidulin): anti-inflammatory;<br>Pectin Polysaccharide II (PMII) antibacterial
	Leaves (applied raw)	Med	Skin diseases and traumas	Acne, pimples, foruncles, skin abscesses<br>Anti-inflammatory, disinfectant and wound healing<br>Skin tags and warts<br>Insect bites	[2,3,13,15,19,22,127,128,130,205]	[127,128,129,130]	
	Leaves (infusion)	Med	Respiratory tract infections	Asthmatic bronchitis<br>Cough	[13,15,127,130]	[127,129,130]	
	Leaves (applied as poultice)	Med	General condition	Anti-inflammatory<br>Infections	[3,13,15,127]	[127,128,129]	
	Leaves (applied raw)	Vet	All animals	Leaves as wound healing	[13,18,22,127,128,130]	[127,128,129,130]	

							[129]<br><br>(Anti urticaria treatment) Ursolic acid, oleanolic acid and α-linolenic acid: inhibition of COX-2 Luteolin: suppresses leukocyte migration and inhibits mast cell degranulation [130]
				**Polygonaceae**			
Rumex acetosa L. Acetosa	Leaves (eaten raw)	Vet	Cattle	Improves milk taste			
Rumex alpinus L. Slavàzza	Leaves (applied as poultice)	Med	Skin diseases and traumas	Wound healing, wounds	[15,18]	[147] (antibacterial) [148] (antibacterial)	Nepodin (2-Acetil-3-methylnaphthalene-1,8-diol): antibacterial [147,148]
	Leaves (Unknown)	Med	Other	Anti-inflammatory			
	Leaves (applied as poultice)	Med	Circulatory system disorders	Varicose vein	[3,15]		
	Leaves (macerated oil, applied as poultice)	Med	Musculoskeletal system disorders and traumas	Anti-inflammatory for joints, Contusions	[13]		
	Leaves (applied as poultice)	Med	General condition	Soothing			
	Leaves (boiled and eaten)	Vet	Pigs	Boiled leaves	[3,15]		
				**Polypodiaceae**			
Polypodium vulgare L. Feles	Underground organs (roots/bulbs/tuber	Med	Digestive tract disorders	Laxative, intestinal motility Vermifuge	[15,22,137,245,246,247]	[137]	Resins of the rhizome: anthelmintic; glycosides (polydin): laxative

	s/rhizomes) (eaten raw)						[137]
	Underground organs (roots/bulbs/tubers/rhizomes) (cooked and eaten)	Med	General condition	Immunomodulant	[137]		
				**Rosaceae**			
Alchemilla vulgaris L. Alchemilla	Epigeal part (Whole) (infusion)	Med	Digestive tract disorders	Digestive	[27,28,248]		Quercetin-3-O-b-D-glucopyranoside, quercetin-3-O-b-D-rutinoside, quercetin-3-O-a-D-arabinofuranoside, and 3-O-kaempferol-6-O-(p-coumaroyl)-b-D-glucopyranoside: anti-inflammatory [27]
	Epigeal part (Whole) (macerated oil)	Med	Musculoskeletal system disorders and traumas	Muscle Inflammations and pain Hernia	[3]	Anti-inflammatory [28]	
	Epigeal part (Whole) (infusion)	Med	Nervous system disorders	Sedative hypnotic, promotes sleep	[13]		Phenols: anti-inflammatory and antioxidant [28]
Crataegus monogyna Jacq. Biancospino	Flowers/inflorescences/flowering tops (infusion)	Med	Respiratory tract infections	Cough	[249]	[250] Antibacterial	
Prunus avium L. Ciliegio	Fruits/infructescences/accessory fruits (infusion)	Med	Urinary tract disorders	Diuretic, Urinary tract depurative	[2,14,15,138]	[138] (only stalks)	
	Seeds (put in a pillow and heated)	Med	Musculoskeletal system disorders and traumas	Muscle Inflammations and pain Rheumatic pain	[15]	[251] (heat therapy)	
Rosa canina L. Fròsole/Frösuli (false fruits)	Flowers/inflorescences/flowering tops	Med	Other	Depurative	[15]		Medicinal properties of *R. canina* (in particular anti-inflammatory and

	(infusion)						antioxidant activities) due to flavonoids, carotenoids, fatty acids, vitamin C [140]<br><br>Particularly: FAs (linoleic and α-linolenic acids); GOPO® (Galactolipid (2S)-1,2-di-O-[(9Z,12Z,15Z)-octadeca-9,12,15-trienoyl]-3-O−B-D-galactopyranosy glycerol); oleanic, ursolic, and betulinic acids: anti-inflammatory activity [140]
	Leaves (infusion)	Med	Digestive tract disorders	Laxative<br>Promotes digestion	[3,15]		
	Leaves (infusion)	Med	Urinary tract disorders	Depurative	[3,13,139,140]	[139]	
	Flowers/inflorescences/flowering tops (infusion)	Med	Nervous system disorders	Relaxant			
	Fruits/infructescences/accessory fruits (infusion)	Med	Respiratory tract infections	Decongestant<br>Sorethroat	[3,14,15,139,140]	[139,140]	
				**Solanaceae**			
Solanum tuberosum L.<br>Patata	Underground organs (roots/bulbs/tubers/rhizomes) (tubers sliced and applied)	Med	Ophthalmic ailments	Ocular anti-inflammatory	[3,15,205]	Anti-inflammatory [159,160,161]	Anti-inflammatory activity: glycoalkaloids (α-chaconine, α-solanine, and solanidine) [159,160]; isolated peptides [161]<br><br>Antioxidant activity: phenolic compounds (chlorogenic acid), proteins (patatins), and peptides [160]
				**Urticaceae**			
Urtica dioica L.<br>Ortica	Leaves (cooked and eaten)	Med	Circulatory system disorders	Blood depurative	[13,14,174,175]	[173,174]	Anti-inflammatory activity against muscle pain and rheumatism: acetylcholine, serotonine, formic acid [173]; terpenes and phenols [176]
	Leaves (infusion)	Med	Digestive tract disorders	Digestion, Liver depurative	[2,3,15,22,174,206]	[173,174]	
	Leaves (infusion)	Med	Urinary tract disorders	Depurative	[2,3,13,15,18,173,174,175]	[173,174]	

Leaves (applied as a poultice)	Med	Musculoskeletal system disorders and traumas	Rheumatism, Contusions, Hematoma	[3,13,14,15,22,173,174,175,205]	[65,173,174,175,176]
Epigeal part (Whole) (infusion)	Med	General condition	Anti-inflammatory, Corroborant	[174]	[173,174,176]

© 2020 by the authors. Submitted for possible open access publication under the terms and conditions of the Creative Commons Attribution (CC BY) license (http://creativecommons.org/licenses/by/4.0/).
